# A Unified Mass Spectrometry Workflow Integrating Bottom-Up and Native Top-Down Proteomics via Nanodroplet Enzymatic Digestion

**DOI:** 10.1021/acs.analchem.6c01549

**Published:** 2026-06-03

**Authors:** Hsi-Wen Wang, Yong-Lun Hong, Ting-Yi Chiang, Szu-Hsueh Lai

**Affiliations:** Department of Chemistry, 34912 National Cheng Kung University, Tainan TW-701, Taiwan

## Abstract

Bottom-up and native top-down proteomics provide complementary but traditionally disconnected views of protein composition and structure. Integrating these approaches into a single, streamlined workflow has remained challenging due to incompatible sample preparation and analytical requirements. Here we report a unified mass spectrometry platform that bridges bottom-up and native top-down analysis through nanodroplet-accelerated enzymatic digestion performed directly under native electrospray conditions. By tuning the extent of digestion within nanodroplets, intact proteins, protein assemblies, and proteolytic peptides are generated simultaneously and analyzed within a single experiment. This workflow enables simultaneous peptide-level identification and native proteoform analysis within a single mass spectrometric experiment. We demonstrate the generality of this approach using myoglobin, amphipathic β-casein isoforms, and the 800 kDa GroEL chaperonin complex, achieving rapid sequence coverage while preserving native structural information accessible by native top-down mass spectrometry. This integrated strategy provides a practical route for comprehensive proteoform and protein assembly characterization across multiple levels of structural organization.

## Graphical abstract

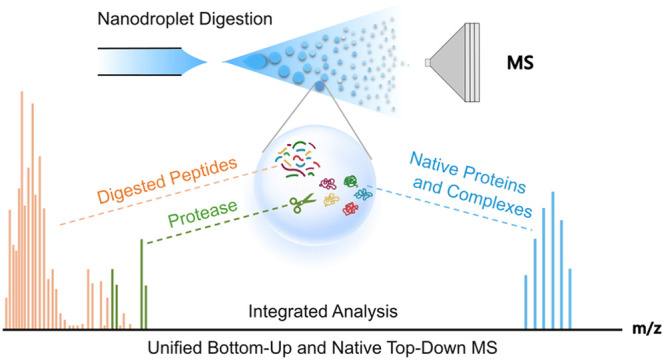

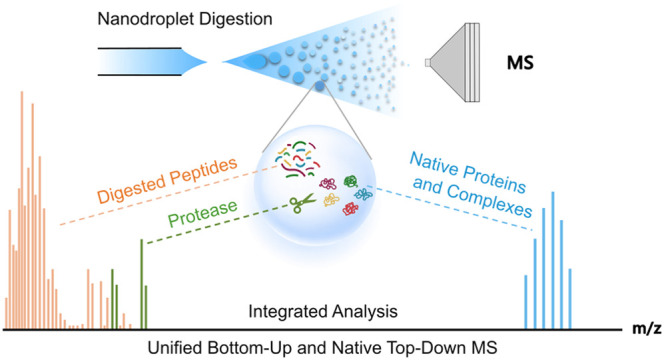

## Introduction

Bottom-up proteomics^1–4^ and native top-down mass spectrometry^5–8^ represent two cornerstone methodologies for protein characterization, each offering complementary analytical strengths. Bottom-up proteomics enables sensitive and high-throughput protein identification through enzymatic digestion followed by peptide-centric mass spectrometric analysis.^2,4^ This approach underpins most large-scale proteomics workflows^3^ and has been widely applied to sequence identification, post-translational modification (PTM) analysis,^9,10^ and quantitative studies of complex biological systems.^11^ However, because proteins are analyzed indirectly through their constituent peptides, bottom-up proteomics inherently sacrifices information on higher-order structure, subunit connectivity, and native protein assemblies.^12^

Native top-down mass spectrometry preserves noncovalent interactions during ionization, enabling direct interrogation of intact proteins, proteoforms, and protein assemblies under native-like conditions.^5–8^ Through controlled gas-phase activation, native top-down methods provide insights into quaternary structure, subunit stoichiometry, ligand binding, and the distribution of PTMs across protein complexes.^7,8^ Recent advances in native mass spectrometry have demonstrated the use of in-source dissociation and pseudo-MS^3^ strategies to probe subunit architecture and obtain sequence information from protein complexes.^13^ However, native top-down MS remains limited by incomplete backbone fragmentation and restricted sequence coverage, which can hinder confident localization of sequence variants, mutation sites, and post-translational modifications across intact proteins and protein assemblies.^14^ In this context, the present strategy complements these methods by enabling sequence generation through controlled enzymatic digestion within nanodroplets, thereby reducing reliance on gas-phase fragmentation.

Because of these complementary strengths and limitations, integrating bottom-up proteomics with native top-down MS into a single, streamlined workflow has been an ongoing objective in analytical mass spectrometry.^15–17^ Existing strategies typically rely on offline or partially integrated approaches, in which intact proteins and digested peptides are generated and analyzed separately or under different solution conditions. Such workflows often require extensive sample handling, prolonged processing times, or denaturing buffers, complicating direct correlation between peptide-level information and measurements of intact proteins or complexes. As a result, connectivity between peptides and their parent proteoforms is usually inferred rather than directly observed.

Droplet-based enzymatic digestion has emerged as a promising strategy to accelerate proteolysis while minimizing sample handling.^18–23^ Previous studies have shown that enzymatic reactions can be dramatically accelerated in microdroplets, enabling protein digestion on millisecond time scales, commonly implemented using electrosonic spray ionization (ESSI).^24^ While highly effective for rapid peptide generation, such methods typically rely on gas-assisted nebulization, large emitter diameters, and/or ammonium bicarbonate buffers, which can induce partial protein unfolding and disrupt noncovalent interactions—conditions generally incompatible with native mass spectrometry.^25^ Consequently, microdroplet digestion has been successfully applied in proteomics but remains challenging to integrate with native mass spectrometry workflows due to compatibility considerations.

Recent studies have shown that nanoESI emitter diameter strongly influences droplet size and reaction kinetics.^26^ Building on microdroplet mass spectrometry using electrosonic spray ionization (ESSI) for enzymatic protein digestion, Li and Ying reported an nESI-based approach to monitor the catalytic process of chymotrypsin under native top-down MS conditions.^27^ While this work represents progress toward implementing enzymatic reactions in the nanodroplet regime, the demonstrated application was limited to a synthetic peptide substrate comprising seven amino acids. Extension of nanodroplet digestion to intact, folded proteins and protein assemblies—particularly under native-compatible conditions—has not been systematically evaluated.

Here, we present a gas-free nanoelectrospray (nanoESI) platform operated in a static configuration, in which nanodroplets are generated without the use of nebulizing or sheath gas, in contrast to gas-assisted microdroplet approaches such as electrosonic spray ionization (ESSI). This configuration enables enzymatic digestion within aqueous nanodroplets under native mass spectrometry conditions. By performing digestion in ammonium acetate and tuning the enzyme-to-protein ratio, this approach generates hybrid ion populations comprising intact proteins, released subunits, and digestion-derived peptides in a single experiment. This configuration enables concurrent bottom-up peptide identification and native top-down analysis under the same solution conditions, without intermediate processing.

We systematically evaluate the analytical performance and information content trade-offs of this unified workflow using three chemically distinct protein classes: the well-characterized model protein myoglobin, the amphipathic and highly phosphorylated β-casein isoforms, and the oligomeric chaperonin GroEL complex. Together, these results establish nanodroplet enzymatic digestion as a practical and versatile strategy for integrating bottom-up and native top-down mass spectrometry within a single analytical platform (Figure 1). Importantly, this workflow is not intended to replace conventional LC–MS/MS-based bottom-up proteomics in high-throughput settings. Instead, it defines a single-process, nanodroplet-enabled analytical regime that prioritizes speed, sensitivity, and preservation of folded-state information accessible by native mass spectrometry. By generating intact proteins or assemblies and digestion-derived peptides within the same measurement, this approach enables direct correlation between peptide-level identifications and native proteoforms under native-compatible conditions—capabilities not accessible using bulk or microdroplet digestion workflows (Table 1).

**Figure 1:**
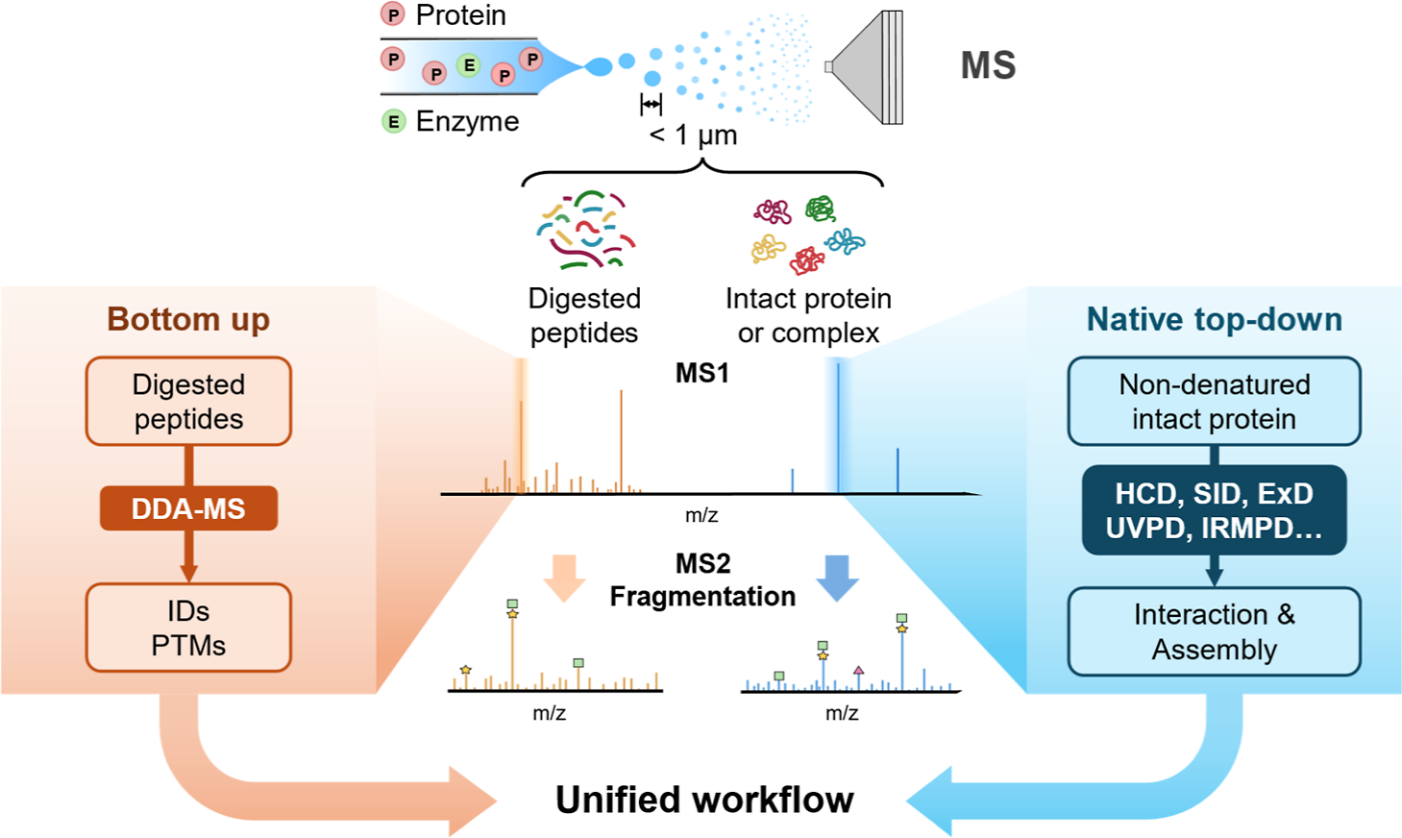
Schematic illustration of the unified nanodroplet enzymatic digestion workflow integrating bottom-up and native top-down mass spectrometry. Proteins and proteases are cointroduced into an aqueous nanoelectrospray emitter, where accelerated yet controllable enzymatic reactions occur within nanodroplets. By tuning the enzyme-to-protein ratio and solution conditions, a hybrid ion population comprising intact proteins or protein assemblies and digestion-derived peptides is generated and directly analyzed by mass spectrometry under native-compatible conditions. This unified ion ensemble enables concurrent peptide-level identification and proteoform- or assembly level characterization within a single experiment.

**Table 1:** Comparison of Representative Proteomic Workflows

workflow	digestion mode and environment	processing time	native proteins/complexes observable	peptide-level protein ID	single-process integration
conventional bottom-up^1–4^	offline, bulk solution	hours	×	×	×
microdroplet digestion^a^ ^,^ ^18,20,23^	online, microdroplet	minutes	×	√	×
**this work** ^b^	**online, nanodroplet** **(native-compatible)**	**minutes**	**√**	**√**	**√**

a Representative microdroplet digestion methods reported in the literature typically employ gas-assisted nebulization and denaturing buffers, limiting compatibility with native mass spectrometry.

b Preserved protein/complex applies to the native top-down component of the workflow; peptide-level information is obtained following enzymatic digestion.

## Results and Discussion

### Ultrafast Nanodroplet Enzymatic Digestion under Native-Compatible Conditions

To evaluate the performance of the online nanodroplet enzymatic digestion platform, myoglobin (accession number: P68082) was selected as a benchmark protein due to its well-characterized structure and extensive use in prior microdroplet digestion studies.^18,28^ Under previously reported microdroplet conditions, near-complete digestion of myoglobin has been achieved using ammonium bicarbonate buffers.^18^ In contrast, the present study employs ammonium acetate, a commonly used solution in native MS, along with reduced protein concentration and low-activity protease, providing a stringent test of digestion efficiency.

Using nanodroplet digestion with direct-injection nanoESI-MS, 41 peptide signals corresponding to 32 unique tryptic peptides were identified, yielding 84% sequence coverage (Figure S1 and Table S1). This performance closely matches reported microdroplet-based digestion results (86% sequence coverage) despite substantially less favorable conditions. Specifically, the protein concentration was reduced 4-fold (2.5 μM versus 10 μM), and a low-activity trypsin preparation (1000–2000 BAEE units mg^–1^) was employed. In addition, ammonium acetate was used instead of ammonium bicarbonate, eliminating bubble-induced unfolding effects associated with CO_2_ generation.^25^

To assess the sensitivity limits of the platform, the myoglobin concentration was further decreased to 0.25 μM, corresponding to a total protein input of less than 1 ng. Under these conditions, 66% sequence coverage was achieved (Table S2), demonstrating effective digestion and peptide detection at submicromolar concentrations with minimal sample consumption. To our knowledge, ultrafast enzymatic digestion with this level of sequence coverage at such low protein amounts has not been previously reported.

For comparison, conventional bulk-phase digestion was performed using heat denaturation followed by overnight tryptic digestion. After 14 h, 12 peptides were detected, corresponding to 52% sequence coverage (Table S3), consistent with prior reports.^18^ Urea-assisted bulk digestion improved coverage to approximately 80% (Table S4) but required additional sample preparation steps and prolonged incubation times. In contrast, nanodroplet digestion achieved comparable or higher sequence coverage within milliseconds, without the need for thermal or chemical denaturation.

Taken together, these results demonstrate that nanodroplet enzymatic digestion enables rapid and efficient peptide generation under native-compatible conditions while maintaining high sensitivity and low sample consumption. This performance establishes nanodroplet digestion as a practical and complementary approach to conventional bulk and microdroplet-based digestion methods for bottom-up proteomic analysis.

### Compatibility of Nanodroplet Digestion with Native Top-Down Mass Spectrometry

The high sensitivity of nanodroplet enzymatic digestion enables precise control over the extent of proteolysis by adjusting the enzyme-to-protein ratio. Under limited digestion conditions, intact proteins coexist with digestion-derived peptides within a single mass spectrum, creating an opportunity to integrate bottom-up and native top-down mass spectrometric analyses without additional sample handling.

Using myoglobin as a model system, controlled nanodroplet digestion generated a hybrid ion population comprising native-like intact protein ions and peptide fragments spanning a broad *m*/*z* range (Figures 2 and S2). To enable efficient peptide identification within this mixed ion population, a customized data-dependent acquisition (DDA) strategy was implemented. This approach enabled confident identification of 13 peptides from 15 high-abundance precursor ions using standard database searching (Table S6).

**Figure 2:**
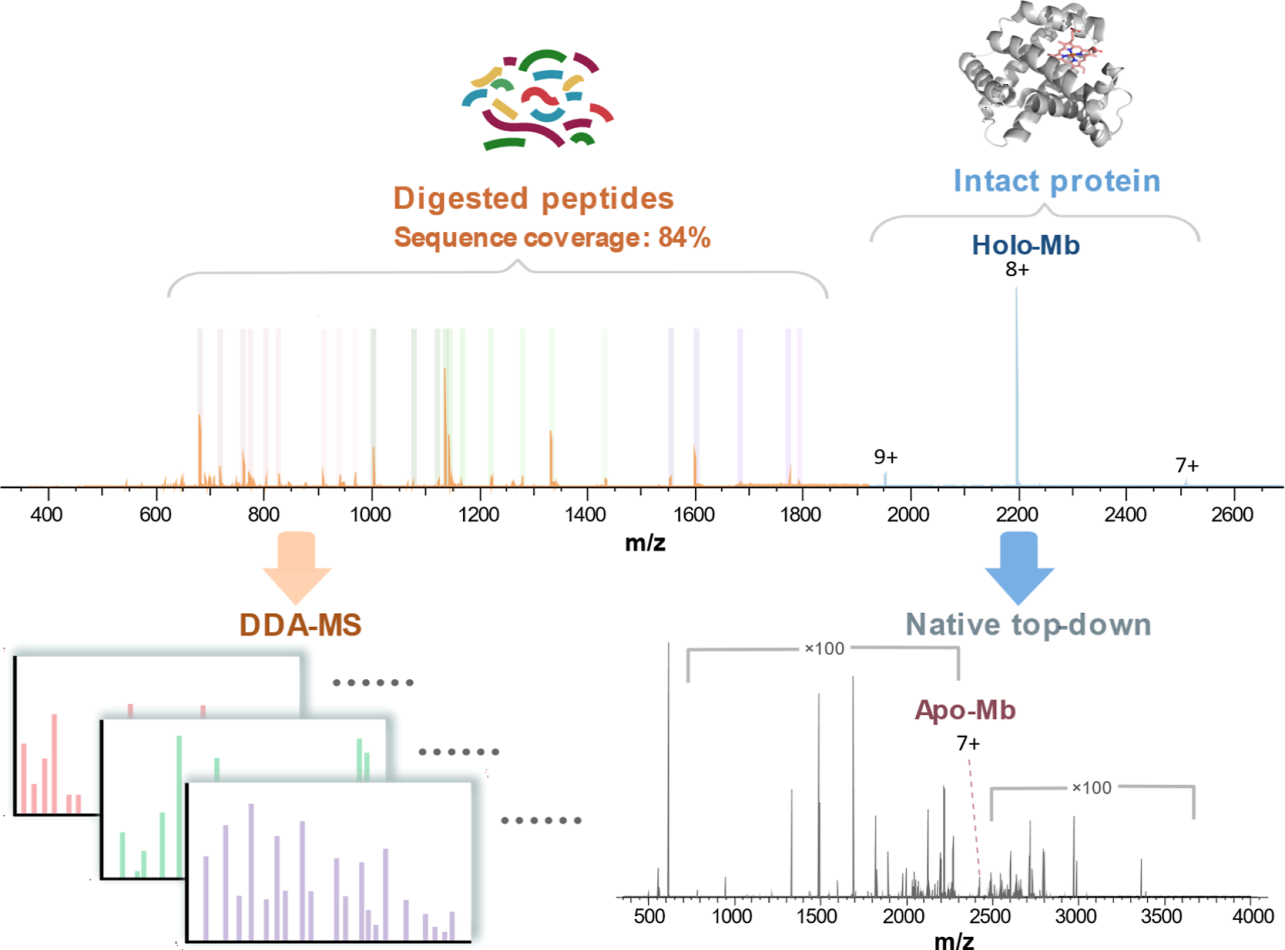
Nanodroplet enzymatic digestion of myoglobin under native conditions. Top panel: Limited digestion of myoglobin (Mb, PDB ID: 1AZI) in nanodroplets yields a spectrum containing both native-like intact protein ions and peptide fragments. Bottom panel: MS^2^ analysis of both precursor ions and their digestion fragments using native top-down and data-dependent acquisition (DDA) methods, respectively. Complete spectral information is provided in Supporting Information Figure S2 and Table S5.

Nanodroplet digestion alone achieved 84% sequence coverage of myoglobin; however, a contiguous internal segment (residues 79–102) remained resistant to enzymatic cleavage. Complementary native top-down analysis was therefore performed on intact myoglobin ions using higher-energy collisional dissociation (HCD). Fragmentation of the intact protein successfully recovered sequence information from this previously inaccessible region, increasing overall sequence coverage to 94% (Figure S3). This result highlights the analytical advantage of integrating bottom-up and native top-down fragmentation within a single experimental platform.

Figure 2 illustrates a representative spectrum acquired under limited digestion conditions, in which native-like intact myoglobin ions coexist with digestion-derived peptides. Reducing the enzyme-to-protein ratio led to the appearance of higher-mass peptide fragments (*m*/*z* 1200–1600), corresponding to peptides up to approximately 7 kDa. These larger fragments exhibited multiple missed cleavages and higher charge states (up to +7), consistent with partial proteolysis. While incomplete cleavage is typically viewed as a limitation in conventional bottom-up workflows, in this context, it does not compromise sequence coverage and instead contributes complementary information accessible through MS^2^ analysis.

Importantly, both peptide- and protein-level MS^2^ experiments were conducted on the same platform without altering experimental conditions. Digestion-derived peptides were fragmented using DDA-based MS^2^, while intact proteins were subjected to native top-down fragmentation by HCD.^11^ This dual capability enables direct correlation between peptide-level identifications and intact protein species within a single measurement, a feature that is difficult to achieve using conventional offline workflows.

Collectively, these results demonstrate that nanodroplet enzymatic digestion can be seamlessly integrated with native top-down mass spectrometry within a single analytical workflow. Before extending this strategy to more challenging protein systems, it is important to clarify the intended scope of this workflow relative to conventional proteomics approaches. Although conventional LC–MS/MS-based bottom-up proteomics remains unparalleled in terms of depth of sequence coverage, proteome-wide throughput, and automated data acquisition, the nanodroplet digestion strategy described here is not designed to compete directly with those workflows. Instead, it defines a distinct analytical regime in which reaction acceleration, minimal sample handling, and native-compatible conditions are prioritized over maximal peptide yield. This trade-off enables access to complementary information that is not available in conventional bottom-up experiments, namely the concurrent observation of intact proteins or assemblies, released subunits, and digestion-derived peptides within a single native-compatible measurement. By collapsing digestion and native MS analysis into a single injection, this approach enables direct correlation between peptide-level identifications and intact proteoforms or protein assemblies without parallel sample preparation or inference across separate experiments. In this context, nanodroplet digestion should be viewed as a targeted, information-efficient strategy for rapid proteoform and assembly level interrogation, rather than as a replacement for established high-throughput bottom-up proteomics workflows.

### Direct Analysis of Protein Isoforms and Post-Translational Modifications

While bottom-up proteomics offers high sensitivity for peptide identification, its effectiveness is often limited for amphipathic proteins due to aggregation-prone behavior, restricted protease accessibility, and enzyme-dependent cleavage bias.^29^ These challenges complicate isoform discrimination^30^ and compromise the characterization of post-translational modifications (PTMs) at the intact-protein level. To evaluate the performance of the unified nanodroplet digestion platform under such conditions, bovine β-casein (accession number: P02666) was selected as a representative amphipathic protein with multiple genetic variants and phosphorylations.

Prior to enzymatic digestion, native mass spectra confirmed the integrity of intact β-casein, showing no evidence of fragmentation during ion transmission. Three coexisting isoforms—A2 (23,982 Da), A1 (24,022 Da, P67 → H), and I (23,965 Da, M93 → L)—were clearly resolved, each exhibiting a mass shift consistent with five phosphorylations (Figure 3). This intact-level characterization provides an essential reference for subsequent peptide-level analysis.

**Figure 3:**
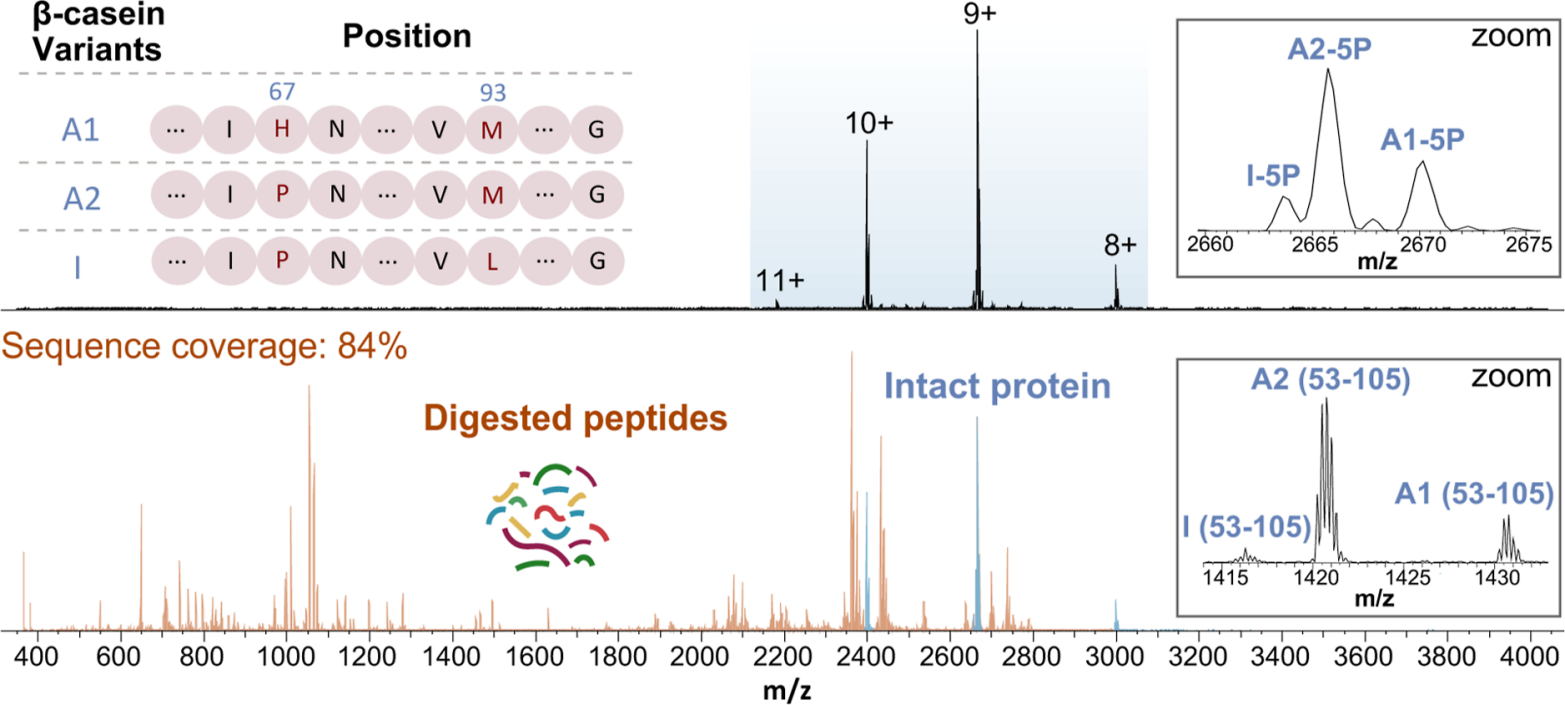
Nanodroplet enzymatic digestion of β-casein using combined trypsin and chymotrypsin on the streamlined platform. Top: Native mass spectra of intact β-casein reveal three coexisting isoforms—A2, A1, and I—each resolved with five endogenous phosphorylations (denoted as—5P). Bottom: Following enzyme addition, partial nanodroplet digestion generates a hybrid ion population comprising intact proteoforms (blue) and digestion-derived peptide fragments (orange). Expanded views highlight isoform-specific peptide signals spanning residues 53–105, corresponding to sequence variants that distinguish A1, A2, and I, and are directly correlated with the intact protein assignments. Complete spectral assignments and peptide identifications are provided in Supporting Information Figure S4 and Table S8.

Conventional enzymatic digestion of β-casein is known to be inefficient due to the presence of extended hydrophobic regions that restrict protease accessibility. Even with prolonged digestion or the use of alternative proteases, reported sequence coverage typically remains below 50%.^31,32^ Consistent with these reports, nanodroplet digestion using trypsin or chymotrypsin alone yielded sequence coverages of 40% and 34%, respectively. However, both enzymes displayed complementary cleavage preferences across the β-casein sequence (Table S7).

By combining trypsin and chymotrypsin within the nanodroplet digestion workflow, sequence coverage increased dramatically to 84%, achieved within milliseconds and without chemical denaturation or extended incubation. This coverage substantially exceeds previously reported values obtained using bulk-phase digestion. Importantly, the high coverage enabled confident identification of isoform-specific peptides spanning residues 53–105, which encompass the sequence variations distinguishing the A1, A2, and I isoforms (Table S8). These assignments were further confirmed by MS^2^ fragmentation (Table S9).

The coexistence of intact proteins and digestion-derived peptides within the same spectrum enables direct correlation between isoform-level assignments and peptide-level sequence information. This feature is particularly valuable for amphipathic proteins, where peptide-only approaches often fail to preserve connectivity between sequence variants and intact proteoforms. Notably, endogenous phosphorylation was preserved under these conditions, supporting PTM analysis alongside intact proteoforms within the same experiment.

In addition to fully cleaved peptides, larger peptide fragments (*m*/*z* ∼2000–2800) were consistently observed near the intact protein signals. These fragments likely arise from incomplete cleavage at regions of reduced protease accessibility and do not compromise sequence coverage. Instead, they may serve as intermediate species that retain partial sequence or structural context, thereby potentially complementing both bottom-up and intact-protein analyses.

Compared with previously reported strategies integrating top-down and bottom-up mass spectrometry via offline fractionation,^33^ the nanodroplet-based approach eliminates the need for extended digestion, chromatographic separation, or separate acquisition modes. For complex proteomic samples, chromatographic separation remains essential, and the present method is positioned for targeted or controlled systems where rapid, integrated structural and compositional information is desired. The ability to resolve intact isoforms, preserve PTMs, and achieve high sequence coverage within a single, rapid measurement highlights the practical advantages of this unified workflow for challenging protein targets. This approach preserves connectivity between intact proteoforms and peptide-level sequence information even for amphipathic and modified proteins.

### Rapid Identification of Protein Assemblies by Integrated Native and Peptide-Level DDA

Large oligomeric protein assemblies present a persistent challenge for mass spectrometry-based analysis due to their high molecular weight, structural heterogeneity, and limited compatibility with conventional bottom-up workflows. In practice, routine characterization of such assemblies often requires separate native MS measurements to confirm intact oligomeric states^34–38^ and independent bottom-up proteomics experiments to establish protein identity, typically involving extended digestion times, chromatographic separation, and manual data integration.^15^

To evaluate whether the unified nanodroplet digestion workflow can streamline analysis of protein assemblies within a single measurement, the tetradecameric chaperonin GroEL (∼800 kDa) was selected as a representative system. Under optimized nanodroplet conditions, native mass spectra preserved the intact GroEL complex, yielding well-resolved charge-state distributions corresponding to the assembled oligomer (Figure 4). No evidence of extensive subunit dissociation or unfolding was observed, indicating that the nanodroplet environment and nanoESI ionization are compatible with large, noncovalent protein assemblies.

**Figure 4:**
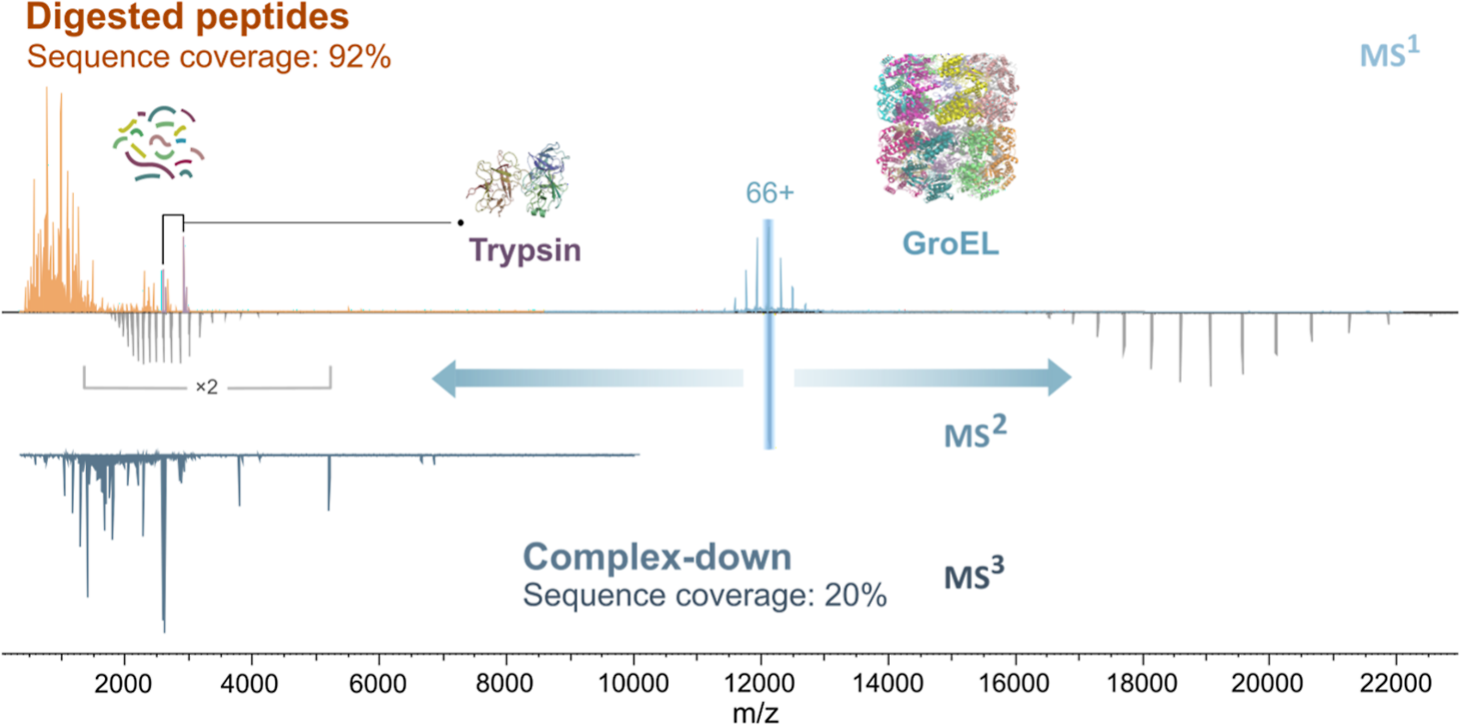
Comprehensive structural and compositional analysis of protein complexes. Top panel: Mass spectrum of GroEL (PDB ID: 1KP8) following nanodroplet tryptic digestion, showing coexisting native-like tetradecamer (14-mer), trypsin (PDB ID: 1AVX), and digested peptides that collectively provide 92% sequence coverage. Bottom panel: MS^2^ spectra of the ejected monomer (left) and 13-mer (right), both derived from precursor tetradecamer ions (highlighted with a blue background) and fragmented using higher-energy collisional dissociation (HCD). The lower spectrum depicts a pseudo-MS^3^ experiment achieved by combining in-source trapping (IST), quadrupole isolation at *m*/*z* 2200 ± 5, and HCD fragmentation of the unfolded monomer, resulting in approximately 20% sequence coverage. Complete spectral information is provided in Supporting Information Figure S5 and Table S10.

In parallel with intact complex detection, peptide ions were generated in the same experiment and subjected to data-dependent acquisition (DDA). Without prior chemical or physical denaturation, GroEL was confidently identified based on the detection of 12 high-abundance proteotypic peptides directly from the nanodroplet-generated ion ensemble (Table S11). Database searching yielded a confident assignment to *Escherichia coli* GroEL (accession number: A1AJ51), demonstrating that peptide-level protein identification can be achieved concurrently with native assembly detection.

This combined readout of intact oligomeric structure and sequence-level identification was obtained within a single, chromatography-free measurement, designed to prioritize rapid identity confirmation and structural context on a processing time scale of minutes. In contrast, conventional approaches typically require separate native MS and bottom-up proteomics workflows, often extending total analysis times from hours to days.

To assess the practical utility of the platform, we analyzed a sample contaminated with unanticipated protein assemblies that were copurified during Ni-NTA affinity purification of a recombinant protein expressed in *E. coli*. Peptide-level DDA-MS analysis identified ArnA (accession number: B1IXT2), supported by 13 high-abundance peptides (Table S12). At the native MS level, intact ArnA assemblies, including tetrameric and hexameric species, were directly observed with measured masses consistent with the theoretical sequence values. Taken together, native and peptide-level measurements enabled rapid determination of both assembly state and protein identity (Figure 5).

**Figure 5:**
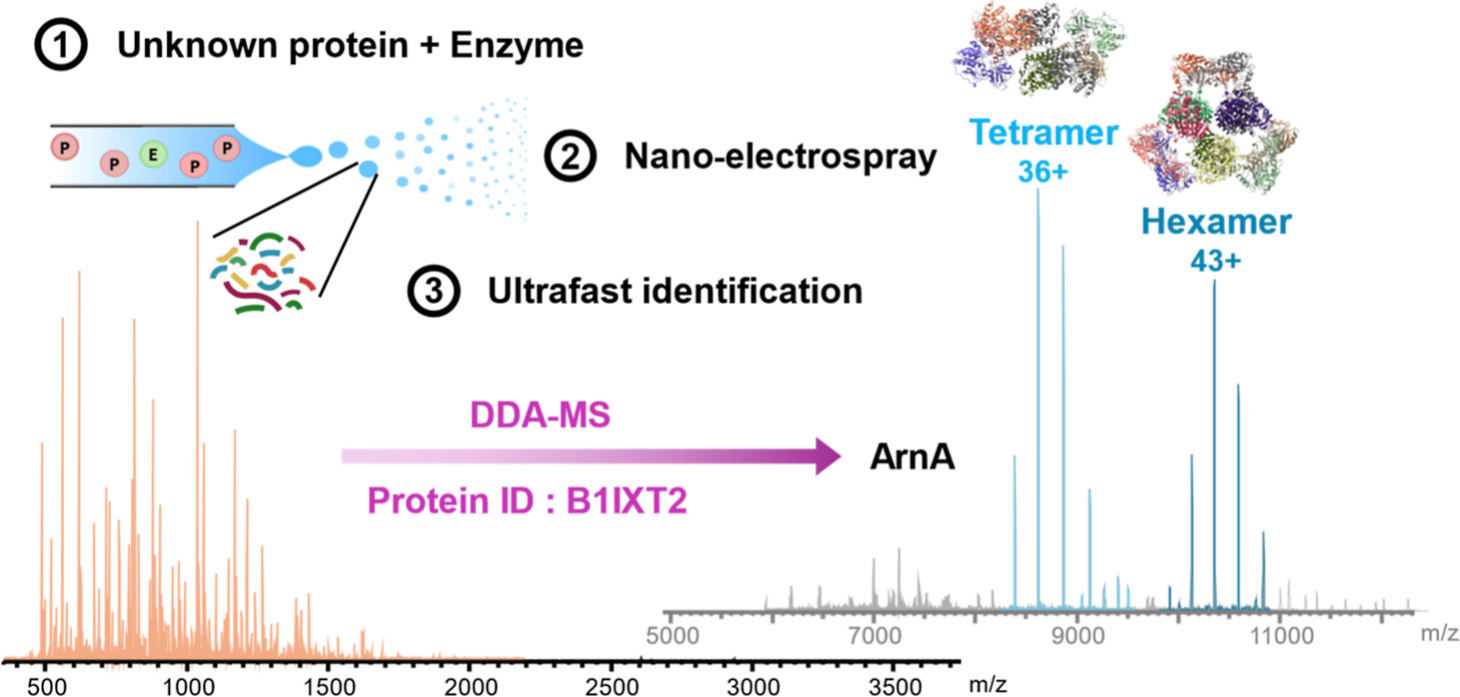
Native MS and peptide-level identification of an unanticipated protein assembly copurified during Ni-NTA affinity purification of a recombinant protein. The peptide-level DDA-MS spectrum (orange) identifies ArnA via proteolytic peptides, while the native MS spectrum (blue) reveals intact tetrameric (PDB ID: 6PIK) and hexameric ArnA assemblies (PDB ID: 6PIH). Complete DDA-MS identification details are provided in Supporting Information Table S12.

In this experiment, both the presence of the assembly and its molecular identity were established without targeted enrichment, prior knowledge of the contaminant, or additional sample preparation. Collectively, these results demonstrate that the unified nanodroplet digestion platform supports rapid, unbiased identification of protein assemblies using native-compatible ionization and peptide-level DDA. The ability to resolve intact oligomeric species and to generate sequence-informative fragments provides a practical analytical solution for protein samples and complexes that require speed, minimal sample handling, and comprehensive molecular characterization.

## Conclusion

In summary, we demonstrate that nanodroplet enzymatic digestion enables a unified mass spectrometry workflow that integrates bottom-up identification with native top-down and complex-level analysis in a single experiment. This approach supports rapid peptide generation while preserving native proteoforms and assemblies, extending from monomeric proteins to large oligomeric complexes. By unifying peptide-level identification and native protein analysis within a single platform, this strategy provides a broadly accessible analytical framework for multilevel protein characterization that is directly compatible with existing high-resolution mass spectrometers.

## Materials and Methods

### Protein Samples

Horse heart myoglobin, β-casein, proteases, and solvents were purchased from Sigma-Aldrich. Myoglobin, β-casein were buffer-exchanged into ammonium acetate using 3 kDa MWCO Amicon Ultra centrifugal filters (Millipore). The GroEL was prepared in-house following established protocols.^39^

ArnA was isolated as a copurifying species during Ni-affinity purification. *E. coli* BL21-CodonPlus-RIPL cells were lysed by French press in Tris-based high-salt lysis buffer containing glycerol and 10 mM imidazole. The lysate was clarified by centrifugation (25,000*g*, 4 °C) and applied to a Ni Sepharose column equilibrated with wash buffer. After washing to baseline, proteins were eluted by step elution with 100 mM imidazole. Fractions were analyzed by SDS-PAGE, and those containing the ∼75 kDa protein were pooled, dialyzed into HEPES-based high-salt storage buffer (with glycerol, EDTA, and DTT), concentrated, and stored at −80 °C. All steps were performed at 4 °C.

### Nanodroplet-MS

Aqueous samples were subsequently mixed with proteases to the following final concentrations for nanodroplet digestion: myoglobin (2.5 μM) with trypsin (0.1 μM) in 5 mM ammonium acetate, unless otherwise stated. β-Casein (2.5 μM) with trypsin (0.1 μM) and chymotrypsin (0.05 μM) in 5 mM ammonium acetate. GroEL protein complex (2.5 μM) with trypsin (1.0 μM) in 150 mM ammonium acetate. Each mixture was then infused into an in-house-prepared capillary, where nanodroplets were generated via nano-ESI using a platinum-coated borosilicate emitter (∼6 μm tip diameter). Importantly, digestion is not controlled by incubation time (Figures S6 and S7) but by enzyme-to-protein ratio (Figures S1 and S2) and droplet-phase reaction kinetics. The resulting ions were introduced into a Q Exactive UHMR mass spectrometer (Thermo Fisher Scientific) for analysis. To balance the relative abundance of digestion-derived peptides, intact proteins, and protein complexes, the enzyme-to-protein ratio can be adjusted together with MS parameters favoring low- or high-*m*/*z* ion transmission, depending on the experimental objective.

Reproducibility of nanodroplet digestion under limited-digestion conditions was evaluated across replicate experiments, yielding consistent peptide identifications, sequence coverage, and coexistence of intact protein signals (Table S13).

The MS inlet capillary was maintained at 275 °C, and the following parameters were typically applied: emitter-inlet distance is about 4 mm, capillary voltage, ∼1.2 kV in positive ion mode with an ion current of ∼100 nA; collision gas, nitrogen; and in-source trapping voltage, 0–1 V, to avoid in-source trapping fragmentation. The HCD voltage was tuned between 1 and 10 V to maximize ion transmission and signal detection while minimizing unintended collision-induced dissociation, as gas-phase fragments are distinct from enzymatically generated peptides. For peptide sequencing, precursor ions were isolated and fragmented by HCD at 25–50 V. MS^1^ and MS^2^ spectra were acquired at resolutions of 12,500 or 25,000 (at *m*/*z* 400), unless otherwise stated. Instrument calibration was performed using CsI clusters across the *m*/*z* range 350–12,000.

The typical data acquisition time in this study was 1–3 min per analysis. Shorter acquisition times (e.g., 10 s) still provided comparable sequence coverage, although with reduced signal-to-noise (S/N) ratios. These results indicate that the method is compatible with rapid analysis while maintaining sufficient sensitivity for peptide detection (Table S14).

### Bulk-phase Protein Digestion

Traditional protein digestion was performed as a reference control using heat- or urea-induced denaturation. For heat denaturation, myoglobin (15 μM, Sigma-Aldrich) was incubated at 95 °C for 5 min, followed by the addition of trypsin (0.5 μM) in 5 mM ammonium acetate (pH 8) and incubation at 37 °C for 14 h; the reaction was quenched by freezing at −20 °C. For urea denaturation, myoglobin (150 μM) was incubated in 8 M urea for 20 min, then urea was removed via buffer exchange using 3 kDa MWCO Amicon Ultra centrifugal filters. The resulting solution was diluted 10-fold and digested with trypsin under the same conditions as above. After digestion, all samples were appropriately diluted and analyzed by nESI-MS.

### Data-Dependent Acquisition (DDA)

DDA analysis was performed by several *m*/*z* segments at 25,000 resolution (*m*/*z* 400), automatic gain control (AGC) 1 × 10^6^, and maximum injection time (IT) 100 ms. Selected peptides were fragmented by HCD (CE 25–50) and MS/MS spectra acquired at 100,000 or 200,000 resolution, AGC 1 × 10^6^, maximum IT 1000 ms, with a loop count of 10.

### Data Processing and Analysis

Following spectral acquisition, .RAW files were converted into.mzML format and deconvoluted using an in-house Python script. We used the MSDeisotope Python library (Joshua Klein, Boston University CBMS) with a minimum score of 10.0 and a mass error tolerance of 0.02 to generate a charge-deconvoluted spectrum, retaining all isotopic peaks.^40,41^ Charge-deconvoluted spectra were then exported as.xlsx files via LCMS-Spectator (Pacific Northwest National Laboratory) for subsequent analysis. Peptide mass assignments were performed using the MS-Bridge tool in Protein Prospector (v6.5.2, University of California, San Francisco, CA, USA). Search parameters included the UniProtKB database, trypsin (and/or chymotrypsin) digestion, a precursor mass tolerance ±20 ppm, and allowance for up to 10 missed cleavages.

For DDA data processing, .RAW files were converted into the.mzML format and then analyzed using FragPipe v22.0 with a precursor mass tolerance of ±30 ppm, while all other parameters were maintained at their default settings. Database searches were performed against the reviewed UniProtKB protein entries. Protein identifications were filtered at a false discovery rate (FDR) of 1% using a target-decoy strategy. In addition, individual peptide identification confidence was evaluated using posterior probability scores derived from statistical modeling, and only peptides with probability greater than 0.99 were retained as high-confidence identifications.

All native top-down and MS/MS data in this study were analyzed using LCMS-Spectator, developed by the Pacific Northwest National Laboratory (PNNL).^42^ For CID fragment comparison, precursor and product ion tolerances were set to 10 ppm. The minimum signal-to-noise (S/N) threshold for filtering was 1.5, and the Pearson correlation threshold for ions was 0.7. All other parameters were kept at their default values.

## Supplementary Material



## Notes

Supplementary figures of nanodroplet digestion mass spectra, incubation-time-dependent control experiments, and native top-down fragmentation (Figures S1–S7); supplementary tables of peptide assignments, sequence coverage, DDA-MS identifications, MS/MS characterization, digestion reproducibility, and acquisition-time-dependent analytical performance for myoglobin, β-casein, GroEL, and ArnA (Tables S1–S14) (PDF)
