# Supplementary material for: A Unified Mass Spectrometry Workflow Integrating Bottom-Up and Native Top-Down Proteomics via Nanodroplet Enzymatic Digestion
Source: Anal Chem. 2026 Jun 3;98(23):17269–77. doi: 10.1021/acs.analchem.6c01549 (PMC13276839; doi:10.1021/acs.analchem.6c01549)
Supplement: Supplementary file 1 [file ac6c01549_si_001.pdf]

Supporting Information

A Unified Mass Spectrometry Workflow Integrating Bottom-Up and Native Top-Down Proteomics via Nanodroplet Enzymatic Digestion

Hsi-Wen Wang[1]; Yong-Lun Hong[1]; Ting-Yi Chiang[1] and Szu-Hsueh Lai[1,*]

[1] Department of Chemistry, National Cheng Kung University, Tainan TW-701, Taiwan.

*Corresponding author: Szu-Hsueh Lai (shlai@gs.ncku.edu.tw)

Table of Contents

Figure S1 3

Figure S2 4

Figure S3 5

Figure S4 6

Figure S5 7

Figure S6. 8

Figure S7. 9

Table S1 10

Table S2 11

Table S3 12

Table S4 13

Table S5 14

Table S6 15

Table S7 16

Table S8 17

Table S9 18

Table S10 19

Table S11 21

Table S12 22

Table S13 23

Table S14 24

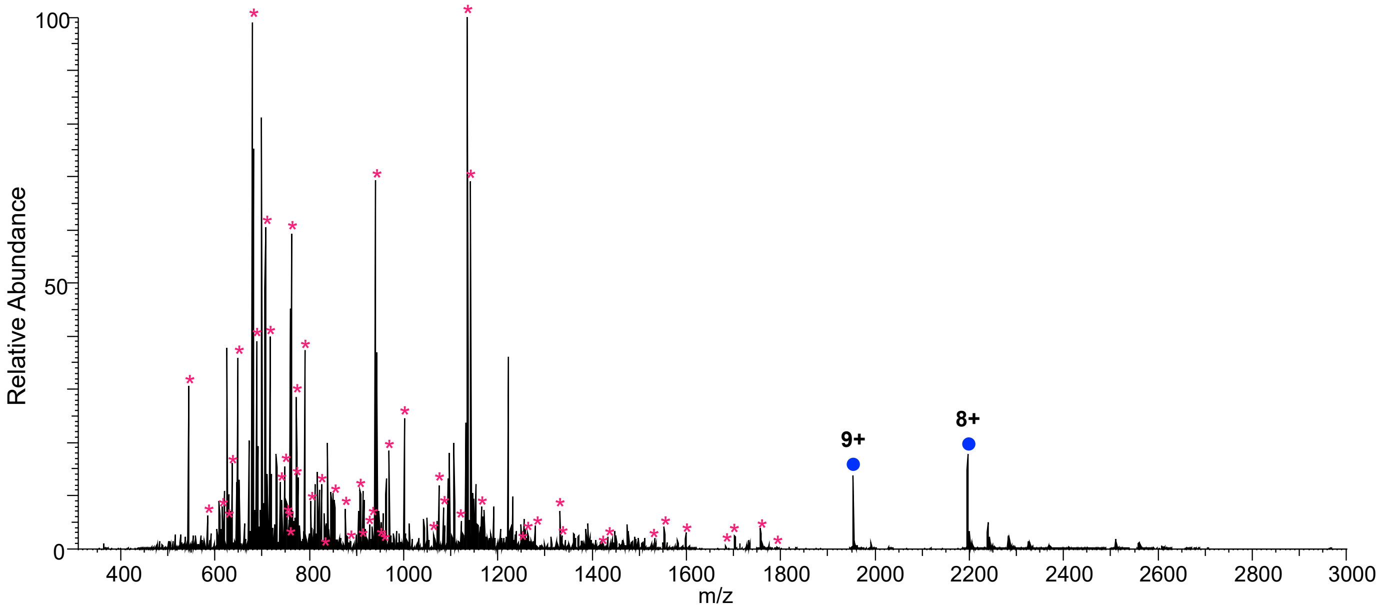

Figure S1 Mass spectrum of peptide fragments from the digestion of 2.5 μM myoglobin with 0.5 μM trypsin in 5 mM ammonium acetate (NH_4OAc) using the nanodroplet platform. Identified peptide peaks are denoted by magenta asterisks, and the intact myoglobin peaks are marked with blue circles.

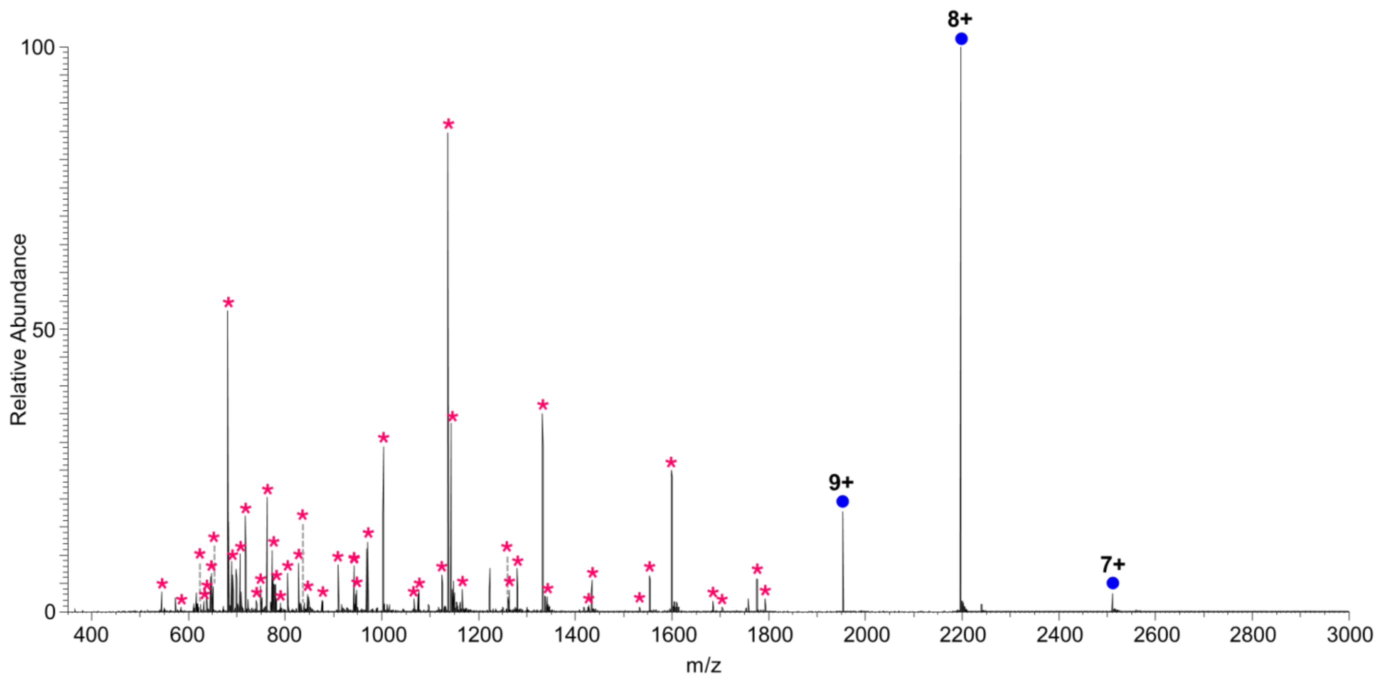

Figure S2 Mass spectrum of peptide fragments from the limited digestion of 2.5 µM myoglobin with 0.1 µM trypsin in 5 mM ammonium acetate (NH_4OAc) using the nanodroplet platform. Identified peptide peaks are denoted by magenta asterisks, and the intact myoglobin peaks are marked with blue circles.

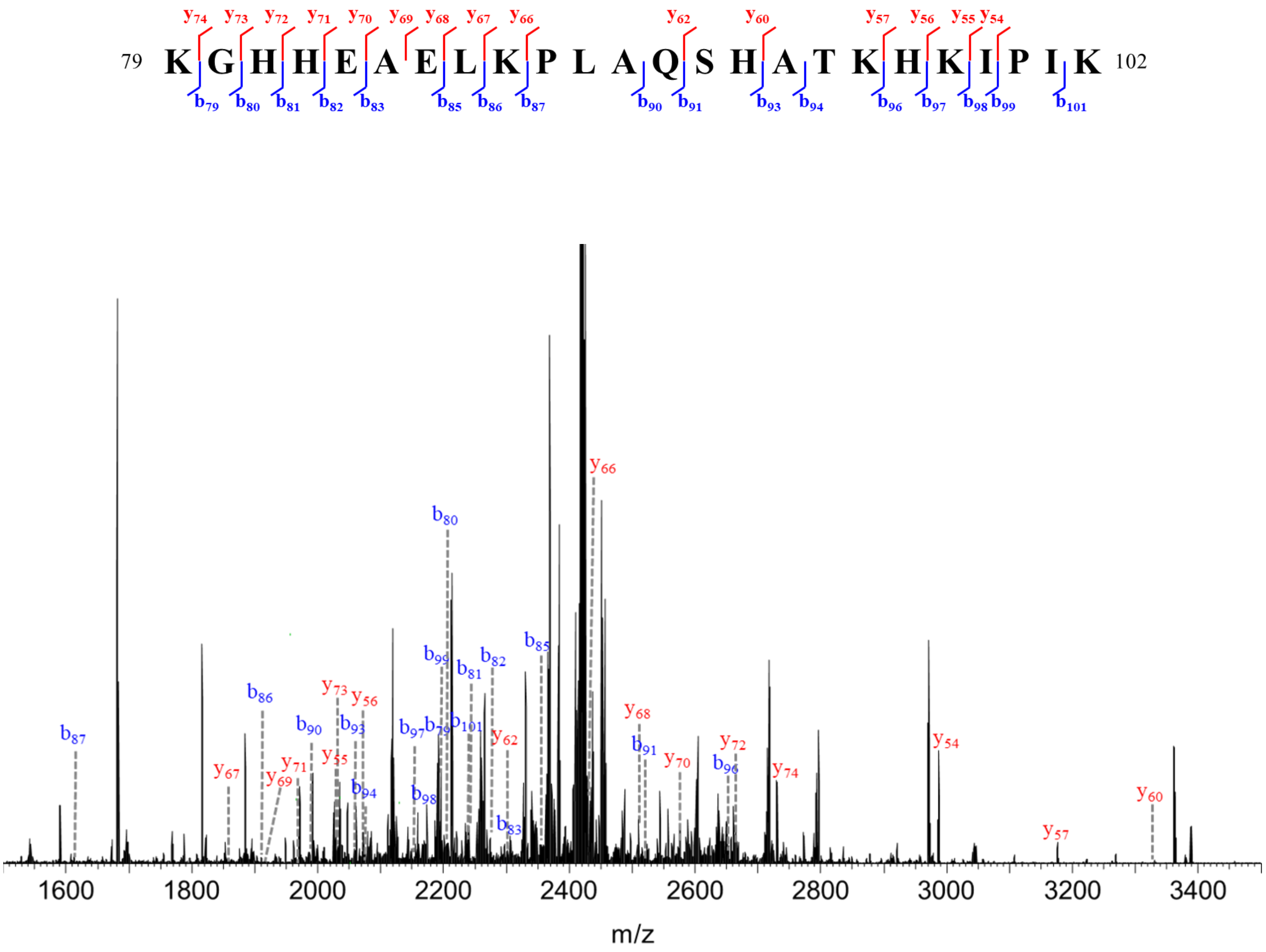

Figure S3 Native top-down fragments of holo-myoglobin by higher-energy collisional dissociation (HCD) recover residues 79–102 missed by bottom-up analysis. The identified residues increased the overall sequence coverage to 94% (144 out of 153 residues)

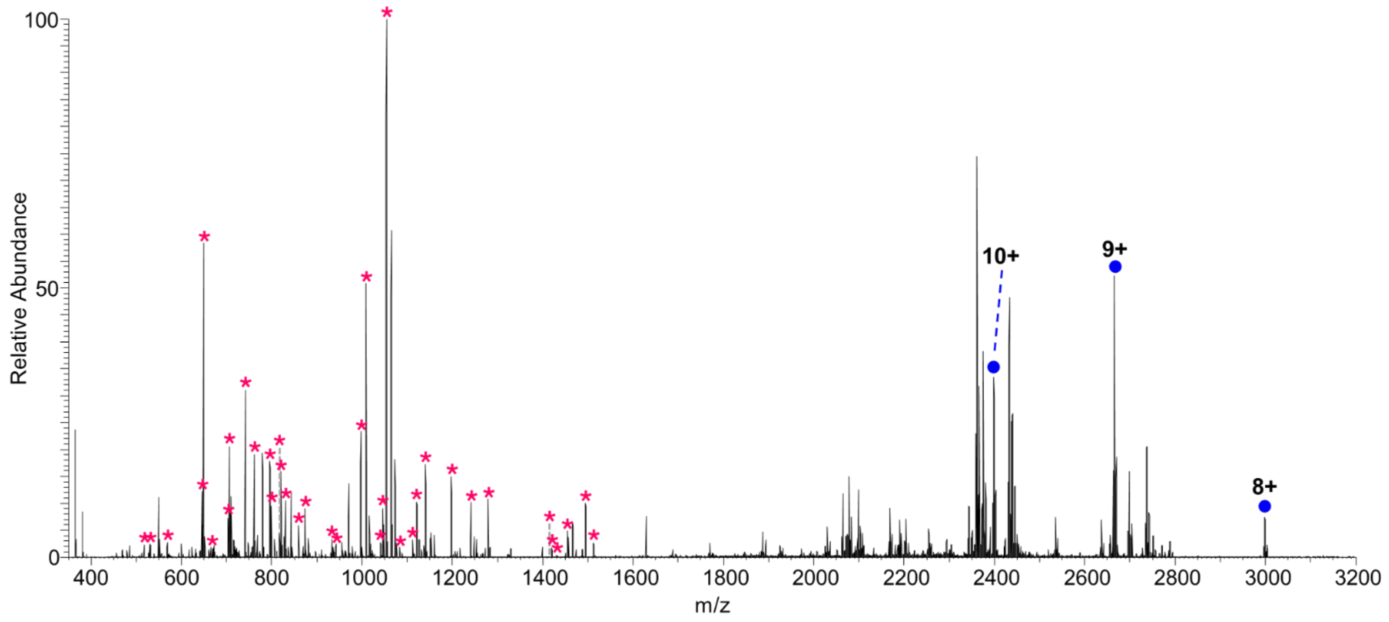

Figure S4 Mass spectrum of peptide fragments from the limited digestion of 2.5 μM β-casein with 0.1 μM trypsin and 0.05 μM chymotrypsin using the nanodroplet platform. Intact β-casein peaks are indicated by blue circles, and identified peptide peaks are highlighted in magenta asterisks.

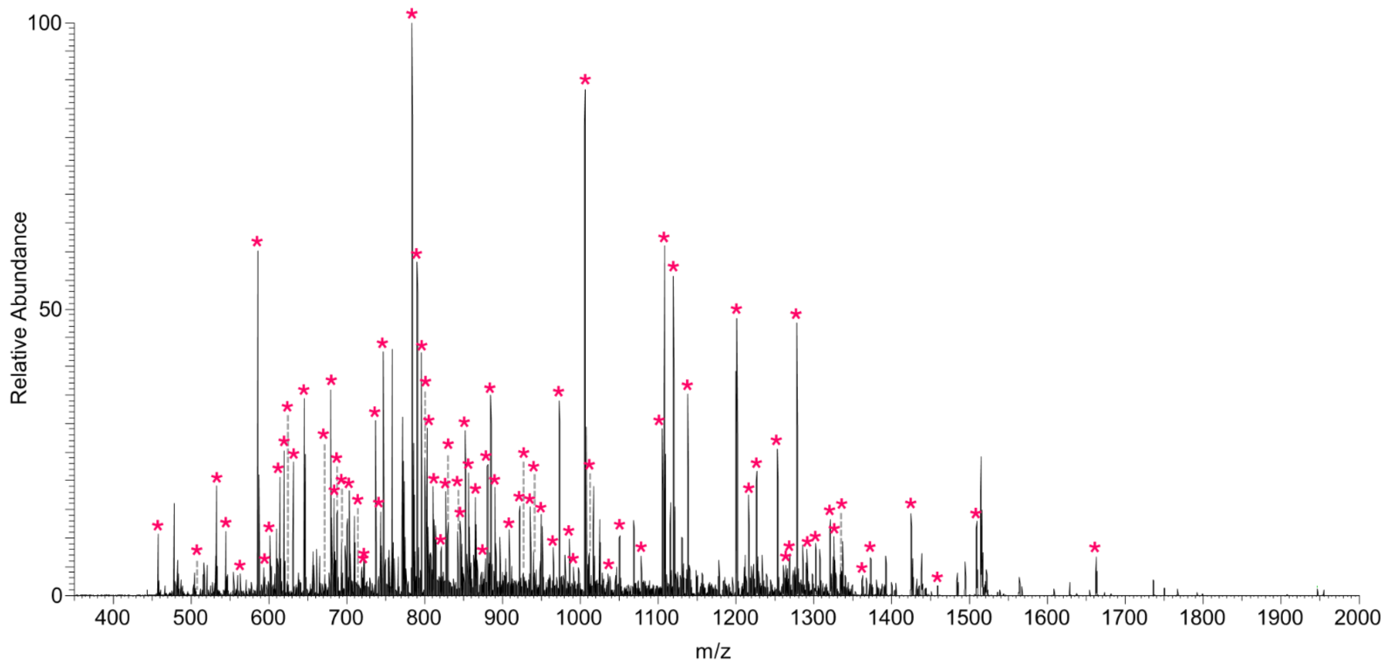

Figure S5 Mass spectrum of peptide fragments from the limited digestion of 2.5 μM GroEL with 1.0 μM trypsin using the nanodroplet platform. Magenta asterisks indicate identified peptide peaks.

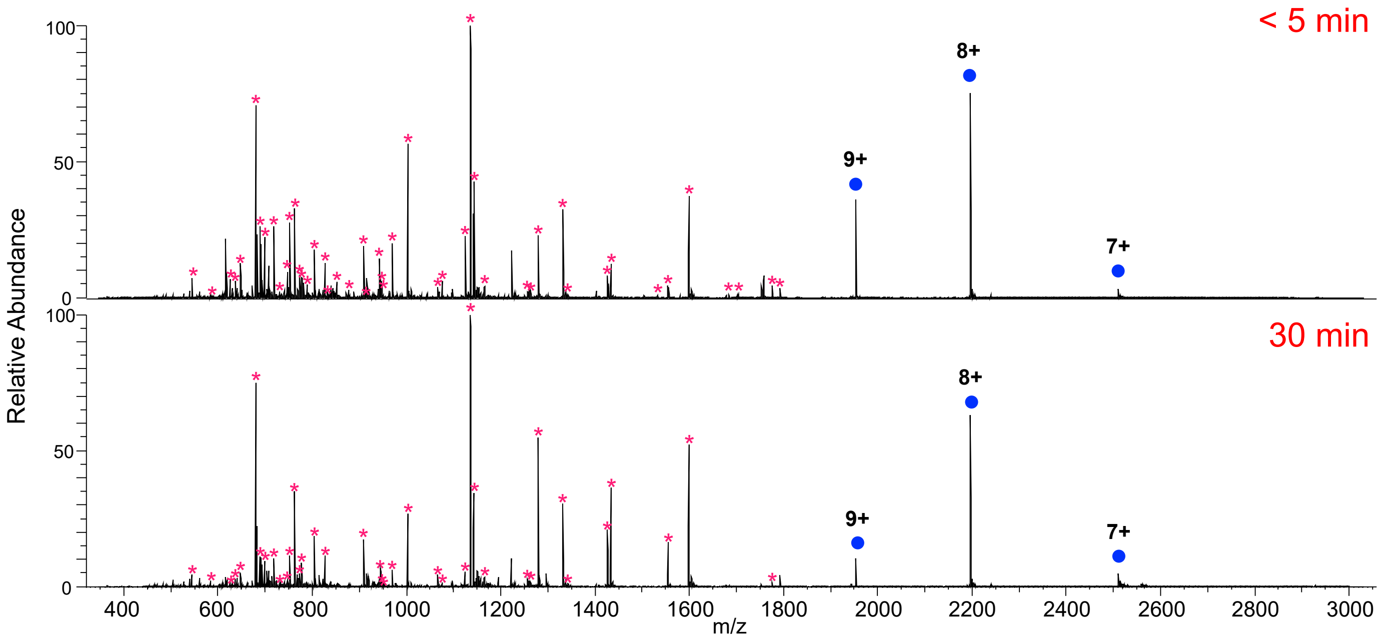

Figure S6 Mass spectra of peptide fragments obtained from limited digestion before (upper) and after (lower) 30 min bulk incubation prior to nanoESI using the nanodroplet platform. Minimal spectral changes following incubation support that proteolysis occurs predominantly during the nanodroplet process rather than in bulk solution. Magenta asterisks indicate identified peptide peaks.

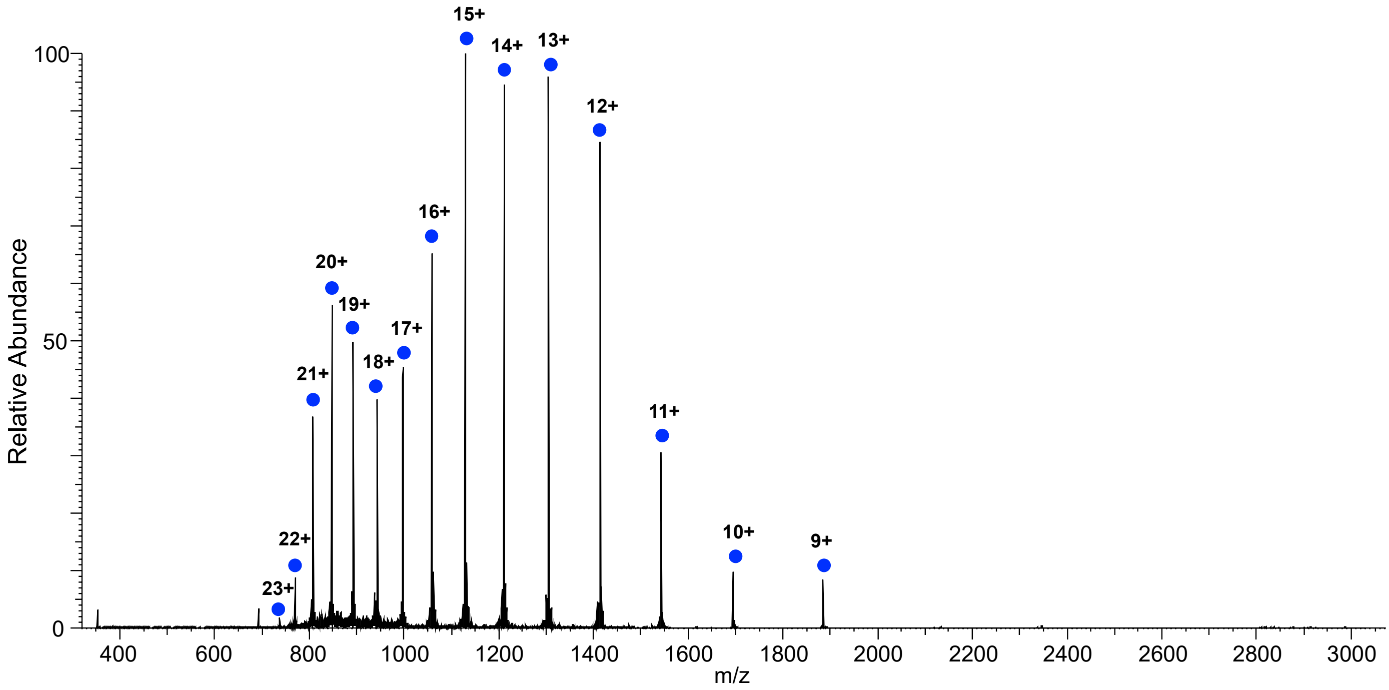

Figure S7 Mass spectrum obtained using the nanodroplet platform after bulk incubation of a concentrated solution containing 5.0 μM myoglobin and 0.2 μM trypsin for 3 h. Following incubation, 1% formic acid was added to terminate proteolysis and denature both the protein and enzyme before nanoESI. No significant peptide formation was observed after bulk incubation, while denatured intact protein signals (marked by blue circles) remained dominant. These results further support that proteolysis occurs predominantly during the nanodroplet process rather than in bulk solution.

Table S1 Peptide fragments from the limited digestion of 2.5 μM Myoglobin with 0.5 μM trypsin using the nanodroplet platform with 5 mM ammonium acetate (NH_4OAc). Detected sequences are highlighted in red, while undetected regions are shown in black. The identified peptides cover 84% of the myoglobin sequence in solution (129 out of 153 amino acids).

1	11	21	31	41	51
GLSDGEWQQV	LNVWGKVEAD	IAGHGQEVLI	RLFTGHPETL	EKFDKFKHLK	TEAEMKASED
61	71	81	91	101	111
LKKHGTVVLT	ALGGILKKKG	HHEAELKPLA	QSHATKHKIP	IKYLEFISDA	IIHVLHSKHP
121	131	141	151		
GDFGADAQGA	MTKALELFRN	DIAAKYKELG	FQG		

Observed peaks (m/z)	Charge state (z)	Observed mass (Da)	Theoretical mass (Da)	Missed cleavage	Peptide sequence
650.33	1	649.32	649.31	0	[148-153] (K)ELGFQG(-)
748.45	1	747.44	747.43	0	[134-139] (K)ALELFR(N)
790.44	1	789.44	789.42	1	[57-63] (K)ASEDLKK(H)
941.49	1	940.48	940.46	1	[146-153] (K)YKELGFQG(-)
1086.58	1	1085.57	1085.55	1	[48-56] (K)HLKTEAEMK(A)
543.79	2	1085.57	1085.55	1	[48-56] (K)HLKTEAEMK(A)
636.35	2	1270.68	1270.66	0	[32-42] (R)LFTGHPETLEK(F)
1360.79	1	1359.78	1359.75	1	[134-145] (K)ALELFRNDIAAK(Y)
680.90	2	1359.78	1359.75	1	[134-145] (K)ALELFRNDIAAK(Y)
689.94	2	1377.86	1377.83	0	[64-77] (K)HGTVVLTALGGILK(K)
740.38	2	1478.75	1478.73	2	[51-63] (K)TEAEMKASEDLKK(H)
753.98	2	1505.95	1505.93	1	[63-77] (K)KHGTVVLTALGGILK(K)
753.98	2	1505.95	1505.93	1	[64-78] (K)HGTVVLTALGGILKK(K)
777.41	2	1552.81	1552.79	2	[140-153] (R)NDIAAKYKELGFQG(-)
803.95	2	1605.87	1605.85	0	[17-31] (K)VEADIAGHGQEVLIR(L)
826.48	2	1650.94	1650.91	2	[134-147] (K)ALELFRNDIAAKYK(E)
831.45	2	1660.87	1660.85	1	[32-45] (R)LFTGHPETLEKFDK(F)
876.48	2	1750.94	1750.91	3	[43-56] (K)FDKFKHLKTEAEMK(A)
584.65	3	1750.94	1750.91	3	[43-56] (K)FDKFKHLKTEAEMK(A)
908.47	2	1814.93	1814.89	0	[1-16] (-)GLSDGEWQQVLNVWGK(V)
620.01	3	1857.00	1856.97	3	[48-63] (K)HLKTEAEMKASEDLKK(H)
969.03	2	1936.04	1936.01	2	[32-47] (R)LFTGHPETLEKFDKFK(H)
646.36	3	1936.04	1936.01	2	[32-47] (R)LFTGHPETLEKFDKFK(H)
1075.65	2	2149.29	2149.25	2	[57-77] (K)ASEDLKKHGTVVLTALGGILK(K)
717.44	3	2149.29	2149.25	2	[57-77] (K)ASEDLKKHGTVVLTALGGILK(K)
760.13	3	2277.38	2277.34	3	[57-78] (K)ASEDLKKHGTVVLTALGGILKK(K)
1142.13	2	2282.25	2282.21	3	[134-153] (K)ALELFRNDIAAKYKELGFQG(-)
761.76	3	2282.25	2282.21	3	[134-153] (K)ALELFRNDIAAKYKELGFQG(-)
947.21	3	2838.60	2838.55	3	[51-77] (K)TEAEMKASEDLKKHGTVVLTALGGILK(K)
989.91	3	2966.71	2966.65	4	[51-78] (K)TEAEMKASEDLKKHGTVVLTALGGILKK(K)
1002.21	3	3003.61	3003.55	4	[32-56] (R)LFTGHPETLEKFDKFKHLKTEAEMK(A)
1123.59	3	3367.73	3367.67	1	[103-133] (K)YLEFISDAIIHVLHSKHPGDFGADAQGAMTK(A)
1702.39	2	3402.77	3402.73	1	[1-31] (-)GLSDGEWQQVLNVWGKVEADIAGHGQEVLIR(L)
1135.27	3	3402.80	3402.73	1	[1-31] (-)GLSDGEWQQVLNVWGKVEADIAGHGQEVLIR(L)
1256.30	3	3765.87	3765.86	4	[119-153] (K)HPGDFGADAQGAMTKALELFRNDIAAKYKELGFQG(-)
1552.84	3	4655.49	4655.38	2	[1-42] (-)GLSDGEWQQVLNVWGKVEADIAGHGQEVLIRLFTGHPETLEK(F)
1164.87	4	4655.45	4655.38	2	[1-42] (-)GLSDGEWQQVLNVWGKVEADIAGHGQEVLIRLFTGHPETLEK(F)
1262.41	4	5045.62	5045.57	3	[1-45] (-)GLSDGEWQQVLNVWGKVEADIAGHGQEVLIRLFTGHPETLEKFDK(F)
1331.22	4	5320.84	5320.73	4	[1-47] (-)GLSDGEWQQVLNVWGKVEADIAGHGQEVLIRLFTGHPETLEKFDKFK(H)
1598.08	4	6388.30	6388.27	6	[1-56] (-)GLSDGEWQQVLNVWGKVEADIAGHGQEVLIRLFTGHPETLEKFDKFKHLKTEAEMK(A)
1278.66	5	6388.25	6388.27	6	[1-56] (-)GLSDGEWQQVLNVWGKVEADIAGHGQEVLIRLFTGHPETLEKFDKFKHLKTEAEMK(A)

Note：”-” means the sequence terminus.

Table S2 Peptide fragments of 0.25 μM myoglobin digested with 0.05 μM trypsin using the nanodroplet platform. Detected sequences are highlighted in red, while undetected regions are shown in black. The identified peptides cover 66% of the myoglobin sequence (101 out of 153 amino acids).

1	11	21	31	41	51
**GLSDGEWQQV**	**LNVWGKVEAD**	**IAGHGQEVLI**	**RLFTGHPETL**	**EKFDKFKHLK**	**TEAEMKASED**
61	71	81	91	101	111
**LKKHGTVVLT**	**ALGGILKKKG**	**HHEAELKPLA**	**QSHATKHKIP**	**IKYLEFISDA**	**IIHVLHSKHP**
121	131	141	151		
**GDFGADAQGA**	**MTKALELFRN**	**DIAAKYKELG**	**FQG**		

Observed peaks (m/z)	Charge state (z)	Observed mass (Da)	Theoretical mass (Da)	Missed cleavage	Peptide sequence
650.32	1	649.32	649.31	0	[148-153] (K)ELGFQG(-)
662.34	1	661.34	661.33	0	[57-62] (K)ASEDLK(K)
941.49	1	940.49	940.46	1	[146-153] (K)YKELGFQG(-)
636.34	2	1270.67	1270.66	0	[32-42] (R)LFTGHPETLEK(F)
680.90	2	1359.78	1359.75	1	[134-145] (K)ALELFRNDIAAK(Y)
777.42	2	1552.82	1552.79	2	[140-153] (R)NDIAAKYKELGFQG(-)
803.95	2	1605.88	1605.85	0	[17-31] (K)VEADIAGHGQEVLIR(L)
818.33	2	1634.00	1634.02	2	[63-78] (K)KHGTVVLTALGGILKK(K)
826.48	2	1650.94	1650.91	2	[134-147] (K)ALELFRNDIAAKYK(E)
908.48	2	1814.93	1814.89	0	[1-16] (-)GLSDGEWQQVLNVWGK(V)
927.45	2	1852.88	1852.95	0	[80-96] (K)GHHEAELKPLAQSHATK(H)
1142.14	2	2282.25	2282.21	3	[134-153] (K)ALELFRNDIAAKYKELGFQG(-)
761.76	3	2282.26	2282.21	3	[134-153] (K)ALELFRNDIAAKYKELGFQG(-)

Note：”-” means the sequence terminus.

Table S3 Peptide fragments of myoglobin digested with trypsin after conventional heat denaturation. Detected sequences are highlighted in red, while undetected regions are shown in black. The identified peptides cover 52% of the myoglobin sequence (80 out of 153 amino acids).

1	11	21	31	41	51
GLSDGEWQQV	LNVWGKVEAD	IAGHGQEVLI	RLFTGHPETL	EKFDKFKHLK	TEAEMKASED
61	71	81	91	101	111
LKKHGTVVLT	ALGGILKKKG	HHEAELKPLA	QSHATKHKIP	IKYLEFISDA	IIHVLHSKHP
121	131	141	151		
GDFGADAQGA	MTKALELFRN	DIAAKYKELG	FQG		

Observed peaks (m/z)	Charge state (z)	Observed mass (Da)	Theoretical mass (Da)	Missed cleavage	Peptide sequence
631.34	1	630.35	630.33	0	[141-145] (R)NDIAAK(Y)
636.34	2	1270.69	1270.66	0	[32-42] (R)LFTGHPETLEK(F)
650.32	1	649.32	649.31	0	[148-153] (K)ELGFQG(-)
708.33	1	707.33	707.32	0	[51-56] (K)TEAEMK(A)
735.49	1	734.49	734.48	1	[97-102] (K)HKIPIK(Y)
748.44	1	747.44	747.43	0	[134-139] (K)ALELFR(N)
751.84	2	1501.68	1501.66	0	[119-133] (K)HPGDFGADAQGAMTK(A)
790.43	1	789.43	789.42	1	[57-63] (K)ASEDLKK(H)
803.93	2	1605.89	1605.85	0	[17-31] (K)VEADIAGHGQEVLIR(L)
941.48	1	940.49	940.47	1	[146-153] (K)YKELGFQG(-)
1271.64	1	1270.64	1270.66	0	[32-42] (R)LFTGHPETLEK(F)
1502.68	1	1501.68	1501.66	0	[119-113] (K)HPGDFGADAQGAMTK(A)

Note：”-” means the sequence terminus.

Table S4 Peptide fragments of myoglobin digested with trypsin after conventional denaturation by urea. Detected sequences are highlighted in red, while undetected regions are shown in black. The identified peptides cover 80% of the myoglobin sequence (123 out of 153 amino acids).

1	11	21	31	41	51
GLSDGEWQQV	LNVWGKVEAD	IAGHGQEVLI	RLFTGHPETL	EKFDKFKHLK	TEAEMKASED
61	71	81	91	101	111
LKKHGTVVLT	ALGGILKKKG	HHEAELKPLA	QSHATKHKIP	IKYLEFISDA	IIHVLHSKHP
121	131	141	151		
GDFGADAQGA	MTKALELFRN	DIAAKYKELG	FQG		

Observed peaks (m/z)	Charge state (z)	Observed mass (Da)	Theoretical mass (Da)	Missed cleavage	Peptide sequence
631.35	1	630.34	630.33	0	[140-145] (R)NDIAAK(Y)
650.32	1	649.31	649.31	0	[148-153] (K)ELGFQG(-)
684.38	1	683.37	683.36	1	[43-47] (K)FDKFK(H)
708.33	1	707.32	707.32	0	[51-56] (K)TEAEMK(A)
735.50	1	734.49	734.48	1	[97-102] (K)HKIPIK(Y)
748.45	1	747.44	747.43	0	[134-139] (K)ALELFR(N)
790.44	1	789.43	789.42	1	[57-63] (K)ASEDLKK(H)
941.49	1	940.48	940.46	1	[146-153] (K)YKELGFQG(-)
1086.58	1	1085.56	1085.55	1	[48-56] (K)HLKTEAEMK(A)
1271.69	1	1270.67	1270.66	0	[32-42] (R)LFTGHPETLEK(F)
636.34	2	1270.68	1270.66	0	[32-42] (R)LFTGHPETLEK(F)
1502.69	1	1501.68	1501.66	0	[119-133] (K)HPGDFGADAQGAMTK(A)
751.85	2	1501.68	1501.66	0	[119-133] (K)HPGDFGADAQGAMTK(A)
1606.88	1	1605.87	1605.85	0	[17-31] (K)VEADIAGHGQEVLIR(L)
803.95	2	1605.87	1605.85	0	[17-31] (K)VEADIAGHGQEVLIR(L)
831.44	2	1660.87	1660.85	1	[32-45] (R)LFTGHPETLEKFDK(F)
927.50	2	1852.98	1852.95	0	[80-96] (K)GHHEAELKPLAQSHATK(H)
618.67	3	1852.98	1852.95	0	[80-96] (K)GHHEAELKPLAQSHATK(H)
991.55	2	1981.07	1981.05	1	[79-96] (K)KGHHEAELKPLAQSHATK(H)
661.37	3	1981.08	1981.05	1	[79-96] (K)KGHHEAELKPLAQSHATK(H)
1055.60	2	2109.17	2109.14	2	[78-96] (K)KKGHHEAELKPLAQSHATK(H)
704.07	3	2109.18	2109.14	2	[78-96] (K)KKGHHEAELKPLAQSHATK(H)
1135.27	3	3402.78	3402.73	1	[1-31] (-)GLSDGEWQQVLNVWGKVEADIAGHGQEVLIR(L)

Note：”-” means the sequence terminus.

Table S5 Peptide fragments from the limited digestion of 2.5 μM myoglobin with 0.1 μM trypsin using the nanodroplet platform. Detected sequences are highlighted in red, while undetected regions are shown in black. The identified peptides cover 84% of the myoglobin sequence (129 out of 153 amino acids).

1<br>GLSDGEWQQV	11<br>LNVWGKVEAD	21<br>IAGHGQEVLI	31<br>RLFTGHPETL	41<br>EKFDKFKHLK	51<br>TEAEMKASED
61<br>LKKHGTVVLT	71<br>ALGGILKKKG	81<br>HHEAELKPLA	91<br>QSHATKHKIP	101<br>IKYLEFISDA	111<br>IIHVLHSKHP
121<br>GDFGADAQGA	131<br>MTKALELFRN	141<br>DIAAKYKELG	151<br>FQG		

Observed peaks (m/z)	Charge state (z)	Observed mass (Da)	Theoretical mass (Da)	Missed cleavage	Peptide sequence
631.35	1	630.35	630.33	0	[140-145] (R)NDIAAK(Y)
650.33	1	649.32	649.31	0	[148-153] (K)ELGFQG(-)
708.33	1	707.32	707.32	0	[51-56] (K)TEAEMK(A)
748.45	1	747.44	747.43	0	[134-139] (K)ALELFR(N)
790.45	1	789.44	789.42	1	[57-63] (K)ASEDLKK(H)
941.49	1	940.49	940.46	1	[146-153] (K)YKELGFQG(-)
543.79	2	1085.57	1085.55	1	[48-56] (K)HLKTEAEMK(A)
636.35	2	1270.68	1270.66	0	[32-42] (R)LFTGHPETLEK(F)
680.90	2	1359.78	1359.75	1	[134-145] (K)ALELFRNDIAAK(Y)
689.94	2	1377.86	1377.83	0	[64-77] (K)HGTVVLTALGGILK(K)
740.39	2	1478.76	1478.73	2	[51-63] (K)TEAEMKASEDLKK(H)
751.85	2	1501.68	1501.66	0	[119-133] (K)HPGDFGADAQGAMTK(A)
777.42	2	1552.82	1552.79	2	[140-153] (R)NDIAAKYKELGFQG(-)
803.95	2	1605.88	1605.85	0	[17-31] (K)VEADIAGHGQEVLIR(L)
826.48	2	1650.94	1650.91	2	[134-147] (K)ALELFRNDIAAKYK(E)
831.45	2	1660.88	1660.85	1	[32-45] (R)LFTGHPETLEKFDK(F)
876.48	2	1750.95	1750.91	3	[43-56] (K)FDKFKHLKTEAEMK(A)
584.66	3	1750.94	1750.91	3	[43-56] (K)FDKFKHLKTEAEMK(A)
908.47	2	1814.93	1814.89	0	[1-16] (-)GLSDGEWQQVLNVWGK(V)
929.51	2	1857.00	1856.97	3	[48-63] (K)HLKTEAEMKASEDLKK(H)
620.01	3	1857.00	1856.97	3	[48-63] (K)HLKTEAEMKASEDLKK(H)
646.36	3	1936.05	1936.01	2	[32-47] (R)LFTGHPETLEKFDKFK(H)
969.03	2	1936.05	1936.01	2	[32-47] (R)LFTGHPETLEKFDKFK(H)
1075.65	2	2149.29	2149.25	2	[57-77] (K)ASEDLKKHGTVVLTALGGILK(K)
717.44	3	2149.30	2149.25	2	[57-77] (K)ASEDLKKHGTVVLTALGGILK(K)
760.14	3	2277.39	2277.34	3	[57-78] (K)ASEDLKKHGTVVLTALGGILKK(K)
1142.14	2	2282.26	2282.21	3	[134-153] (K)ALELFRNDIAAKYKELGFQG(-)
761.76	3	2282.26	2282.21	3	[134-153] (K)ALELFRNDIAAKYKELGFQG(-)
772.43	3	2314.27	2314.25	3	[32-50] (R)LFTGHPETLEKFDKFKHLK(T)
947.21	3	2838.62	2838.55	3	[51-77] (K)TEAEMKASEDLKKHGTVVLTALGGILK(K)
1002.21	3	3003.59	3003.55	4	[32-56] (R)LFTGHPETLEKFDKFKHLKTEAEMK(A)
751.91	4	3003.63	3003.55	4	[32-56] (R)LFTGHPETLEKFDKFKHLKTEAEMK(A)
1123.59	3	3367.75	3367.67	1	[103-133] (K)YLEFISDAIIHVLHSKHPGDFGADAQGAMTK(A)
1702.43	2	3402.85	3402.73	1	[1-31] (-)GLSDGEWQQVLNVWGKVEADIAGHGQEVLIR(L)
1135.29	3	3402.85	3402.73	1	[1-31] (-)GLSDGEWQQVLNVWGKVEADIAGHGQEVLIR(L)
851.71	4	3402.80	3402.73	1	[1-31] (-)GLSDGEWQQVLNVWGKVEADIAGHGQEVLIR(L)
1259.36	3	3775.04	3774.96	6	[32-63] (R)LFTGHPETLEKFDKFKHLKTEAEMKASEDLKK(H)
944.77	4	3775.05	3774.96	6	[32-63] (R)LFTGHPETLEKFDKFKHLKTEAEMKASEDLKK(H)
1531.50	3	4591.47	4591.39	5	[17-56] (K)VEADIAGHGQEVLIRLFTGHPETLEKFDKFKHLKTEAEMK(A)
1552.83	3	4655.47	4655.38	2	[1-42] (-)GLSDGEWQQVLNVWGKVEADIAGHGQEVLIRLFTGHPETLEK(F)
1164.89	4	4655.52	4655.38	2	[1-42] (-)GLSDGEWQQVLNVWGKVEADIAGHGQEVLIRLFTGHPETLEK(F)
932.09	5	4655.43	4655.38	2	[1-42] (-)GLSDGEWQQVLNVWGKVEADIAGHGQEVLIRLFTGHPETLEK(F)
1682.89	3	5045.66	5045.57	3	[1-45] (-)GLSDGEWQQVLNVWGKVEADIAGHGQEVLIRLFTGHPETLEKFDK(F)
1262.43	4	5045.70	5045.57	3	[1-45] (-)GLSDGEWQQVLNVWGKVEADIAGHGQEVLIRLFTGHPETLEKFDK(F)
887.81	6	5320.81	5320.73	4	[1-47] (-)GLSDGEWQQVLNVWGKVEADIAGHGQEVLIRLFTGHPETLEKFDKFK(H)
1331.23	4	5320.88	5320.73	4	[1-47] (-)GLSDGEWQQVLNVWGKVEADIAGHGQEVLIRLFTGHPETLEKFDKFK(H)
1774.63	3	5320.88	5320.73	4	[1-47] (-)GLSDGEWQQVLNVWGKVEADIAGHGQEVLIRLFTGHPETLEKFDKFK(H)
1065.19	5	5320.90	5320.73	4	[1-47] (-)GLSDGEWQQVLNVWGKVEADIAGHGQEVLIRLFTGHPETLEKFDKFK(H)
1341.71	4	5362.82	5362.80	7	[17-63] (K)VEADIAGHGQEVLIRLFTGHPETLEKFDKFKHLKTEAEMKASEDLKK(H)
1425.77	4	5699.04	5698.97	5	[1-50] (-)GLSDGEWQQVLNVWGKVEADIAGHGQEVLIRLFTGHPETLEKFDKFKHLK(T)
1598.11	4	6388.40	6388.27	6	[1-56] (-)GLSDGEWQQVLNVWGKVEADIAGHGQEVLIRLFTGHPETLEKFDKFKHLKTEAEMK(A)
1278.68	5	6388.38	6388.27	6	[1-56] (-)GLSDGEWQQVLNVWGKVEADIAGHGQEVLIRLFTGHPETLEKFDKFKHLKTEAEMK(A)
913.64	7	6388.40	6388.27	6	[1-56] (-)GLSDGEWQQVLNVWGKVEADIAGHGQEVLIRLFTGHPETLEKFDKFKHLKTEAEMK(A)
1790.96	4	7159.80	7159.69	8	[1-63] (-)GLSDGEWQQVLNVWGKVEADIAGHGQEVLIRLFTGHPETLEKFDKFKHLKTEAEMKASEDLKK(H)
1432.97	5	7159.82	7159.69	8	[1-63] (-)GLSDGEWQQVLNVWGKVEADIAGHGQEVLIRLFTGHPETLEKFDKFKHLKTEAEMKASEDLKK(H)

Note：”-” means the sequence terminus.

Table S6 Thirteen proteolytic peptides (probability score > 0.99) were identified from 15 high-abundance peaks by DDA-MS using FragPipe, confirming horse myoglobin (accession number: P68082) with 84% sequence coverage.

Peptide	Charges
[2-17] (M)GLSDGEWQQVLNVWGK(V)	2
[2-32] (M)GLSDGEWQQVLNVWGKVEADIAGHGQEVLIR(L)	3
[2-43] (M)GLSDGEWQQVLNVWGKVEADIAGHGQEVLIRLFTGHPETLEK(F)	3
[33-48] (R)LFTGHPETLEKFDKFK(H)	2, 3
[33-51] (R)LFTGHPETLEKFDKFKHLK(T)	3
[33-57] (R)LFTGHPETLEKFDKFKHLKTEAEMK(A)	3
[49-57] (K)HLKTEAEMK(A)	2
[58-78] (K)ASEDLKKHGTVVLTALGGILK(K)	3
[65-78] (K)HGTVVLTALGGILK(K)	2
[104-134] (K)YLEFISDAIHVLHSKHPGDFGADAQGAMTK(A)	3
[120-134] (K)HPGDFGADAQGAMTK(A)	2
[135-146] (K)ALELFRNDIAAK(Y)	2
[135-154] (K)ALELFRNDIAAKYKELGFQG(-)	2, 3

Table S7 Peptide fragments from the limited digestion of 2.5 μM β-casein with (a) 0.1 μM trypsin and (b) 0.1 μM chymotrypsin using the nanodroplet platform. Detected sequences are highlighted in red, while undetected regions are shown in black.

(a) Trypsin digestion (40 % sequence coverage)

1<br>RELEELNVPG	11<br>EIVESLSSSE	21<br>ESITRINKKI	31<br>EKFQSEEQQQ	41<br>TEDELQDKIH	51<br>PFAQTQSLVY
61 .67<br>PFPGPIPNSL	71<br>PQNIPPLTQT	81<br>PVVVPPFLQP	91 .93<br>EVMGVSKVKE	101<br>AMAPKHKEMP	111<br>FPKYPVEPFT
121<br>ESQSLTLTDV	131<br>ENLHLPLPLL	141<br>QSWMHQPHQP	151<br>LPPTVMFPPQ	161<br>SVLSLSQSKV	171<br>LPVPQKAVPY
181<br>PQRDMPIQAF	191<br>LLYQEPVLGP	201<br>VRGPFPIIV			

Observed peaks, m/z	Charge state, z	Observed mass, Da	Theoretical mass, Da	Missed cleavage	Peptide sequence
646.34	1	645.33	645.31	0	[100-105] (K)EAMAPK(H)
742.46	1	741.46	741.44	0	[203-209] (R)GPFPIIV(-)
748.38	1	747.38	747.36	0	[108-113] (K)EMPFPK(Y)
970.58	3	2908.71	2908.59	1	[184-209] (R)DMPIQAFLLYQEPVLGPVRGPFPIIV(-)
1561.65	2	3121.29	3121.26	1	[1-25] (-)RELEELNVPGEIVES(Phospho)LS(Phospho)S(Phospho)S(Phospho)EESITR(I)
1041.43	3	3121.27	3121.26	1	[1-25] (-)RELEELNVPGEIVES(Phospho)LS(Phospho)S(Phospho)S(Phospho)EESITR(I)
1159.84	3	3476.50	3476.48	2	[1-28] (-)RELEELNVPGEIVES(Phospho)LS(Phospho)S(Phospho)S(Phospho)EESITRINK(K)
1202.53	3	3604.57	3604.57	3	[1-29] (-)RELEELNVPGEIVES(Phospho)LS(Phospho)S(Phospho)S(Phospho)EESITRINKK(I)
1241.04	3	3720.08	3720.03	2	[177-209] (K)AVPYPQRDMPIQAFLLYQEPVLGPVRGPFPIIV(-)
931.02	4	3720.05	3720.03	2	[177-209] (K)AVPYPQRDMPIQAFLLYQEPVLGPVRGPFPIIV(-)
1325.95	3	3974.83	3974.80	4	[1-32] (-)RELEELNVPGEIVES(Phospho)LS(Phospho)S(Phospho)S(Phospho)EESITRINKKIEK(F)
1494.86	3	4481.56	4481.51	3	[170-209] (K)VLPVPQKAVPYPQRDMPIQAFLLYQEPVLGPVRGPFPIIV(-)
1121.40	4	4481.57	4481.51	3	[170-209] (K)VLPVPQKAVPYPQRDMPIQAFLLYQEPVLGPVRGPFPIIV(-)
897.31	5	4481.53	4481.51	3	[170-209] (K)VLPVPQKAVPYPQRDMPIQAFLLYQEPVLGPVRGPFPIIV(-)

(b) Chymotrypsin digestion (34 % sequence coverage)

1<br>RELEELNVPG	11<br>EIVESLSSSE	21<br>ESITRINKKI	31<br>EKFQSEEQQQ	41<br>TEDELQDKIH	51<br>PFAQTQSLVY
61 .67<br>PFPGPIPNSL	71<br>PQNIPPLTQT	81<br>PVVVPPFLQP	91 .93<br>EVMGVSKVKE	101<br>AMAPKHKEMP	111<br>FPKYPVEPFT
121<br>ESQSLTLTDV	131<br>ENLHLPLPLL	141<br>QSWMHQPHQP	151<br>LPPTVMFPPQ	161<br>SVLSLSQSKV	171<br>LPVPQKAVPY
181<br>PQRDMPIQAF	191<br>LLYQEPVLGP	201<br>VRGPFPIIV			

Observed peaks, m/z	Charge state, z	Observed mass, Da	Theoretical mass, Da	Missed cleavage	Peptide sequence
647.33	1	646.32	646.33	0	[53-58] (F)AQTQSL(V)
859.50	2	1716.98	1716.99	1	[194-209] (Y)QEPVLGPVRGPFPIIV(-)
997.59	2	1993.16	1993.14	3	[192-209] (L)LYQEPVLGPVRGPFPIIV(-)
1054.13	2	2106.25	2106.22	4	[191-209] (F)LLYQEPVLGPVRGPFPIIV(-)
1141.10	2	2280.18	2280.15	0	[144-163] (W)MHQPHQPLPPTVMFPPQSVL(S)
761.07	3	2280.18	2280.15	0	[144-163] (W)MHQPHQPLPPTVMFPPQSVL(S)
1512.81	2	3023.61	3023.63	1	[164-190] (L)SLSQSKVLPVPQKAVPYPQRDMPIQAF(L)
1046.57	3	3136.69	3136.71	2	[164-191] (L)SLSQSKVLPVPQKAVPYPQRDMPIQAFL(L)

Table S8 Peptide fragments from the limited digestion of 2.5 μM β-casein with 0.1 μM trypsin and 0.05 μM chymotrypsin using the nanodroplet platform. Detected sequences are highlighted in red, while undetected regions are shown in black. The identified peptides provided 84% sequence coverage of each β-casein variant (176 of 209 amino acids).

Variant *A2*

1	11	21	31	41	51
RELEELNVPG	EIVESLSSSE	ESITRINKKI	EKFQSEEQQQ	TEDELQDKIH	PFAQTQSLVY
61 67	71	81	91 93	101	111
PFPGPIPNSL	PQNIPPLTQT	PVVVPPFLQP	EVMGVSKVKE	AMAPKHKEMP	FPKYPVEPFT
121	131	141	151	161	171
ESQSLTLTDV	ENLHLPLPLL	QSWMHQPHQP	LPPTVMFPPQ	SVLSLSQSKV	LPVPQKAVPY
181	191	201			
PQRDMPIQAF	LLYQEPVLGP	VRGPFPIIV			

Variant *A1* (P67→H)

Variant *I* (M93→L)

Observed peaks (m/z)	Charge state (z)	Observed mass (Da)	Theoretical mass (Da)	Missed cleavage	Peptide sequence	Variant
517.33	1	516.33	516.33	1	[29-32] (K)KIEK(F)	
646.33	1	645.32	645.32	0	[100-105] (K)EAMAPK(H)	
649.36	1	648.35	648.35	1	[164-169] (L)SLSQSK(V)	
748.37	1	747.36	747.37	0	[108-113] (K)EMPFPK(Y)	
821.40	1	820.39	820.39	0	[184-190] (R)DMPIQAF(L)	
830.46	1	829.45	829.45	0	[177-183] (K)AVPYPQR(D)	
873.49	1	872.48	872.49	1	[98-105] (K)VKEAMAPK(H)	
934.47	1	933.46	933.47	1	[184-191] (R)DMPIQAFL(L)	
1084.60	1	1083.59	1083.60	0	[78-87] (L)TQTPVVVPPF(L)	
569.82	2	1137.62	1137.64	2	[98-107] (K)VKEAMAPKHK(E)	
705.92	2	1409.83	1409.83	2	[164-176] (L)SLSQSKVLPVPQK(A)	
796.46	2	1590.91	1590.93	1	[170-183] (K)VLPVPQKAVPYPQR(D)	
531.32	3	1590.93	1590.93	1	[170-183] (K)VLPVPQKAVPYPQR(D)	
816.91	2	1631.80	1631.82	1	[177-190] (K)AVPYPQRDMPIQAF(L)	
859.49	2	1716.97	1717.00	2	[194-209] (Y)QEPVLGPVRGPFPIIV(-)	
941.02	2	1880.02	1880.06	3	[193-209] (L)YQEPVLGPVRGPFPIIV(-)	
997.59	2	1993.16	1993.15	4	[192-209] (L)LYQEPVLGPVRGPFPIIV(-)	
665.38	3	1993.11	1993.15	4	[192-209] (L)LYQEPVLGPVRGPFPIIV(-)	
1054.12	2	2106.22	2106.23	5	[191-209] (F)LLYQEPVLGPVRGPFPIIV(-)	
703.09	3	2106.24	2106.23	5	[191-209] (F)LLYQEPVLGPVRGPFPIIV(-)	
1111.64	2	2221.27	2221.27	3	[164-183] (L)SLSQSKVLPVPQKAVPYPQR(D)	
741.42	3	2221.24	2221.27	3	[164-183] (L)SLSQSKVLPVPQKAVPYPQR(D)	
1141.09	2	2280.17	2280.16	0	[144-163] (W)MHQPHQPLPPTVMFPPQSVL(S)	
761.06	3	2280.16	2280.16	0	[144-163] (W)MHQPHQPLPPTVMFPPQSVL(S)	
571.30	4	2281.16	2281.11	3	[114-133] (K)YPVEPFTESQSLTLTDVENL(H)	
1197.65	2	2393.29	2393.30	2	[170-190] (K)VLPVPQKAVPYPQRDMPIQAF(L)	
798.78	3	2393.31	2393.30	2	[170-190] (K)VLPVPQKAVPYPQRDMPIQAF(L)	
1455.30	2	2908.58	2908.60	6	[184-209] (R)DMPIQAFLLYQEPVLGPVRGPFPIIV(-)	
1512.84	2	3023.66	3023.63	4	[164-190] (L)SLSQSKVLPVPQKAVPYPQRDMPIQAF(L)	
1008.88	3	3023.61	3023.63	4	[164-190] (L)SLSQSKVLPVPQKAVPYPQRDMPIQAF(L)	
1041.42	3	3121.24	3121.27	4	[1-25] (-)RELEELNVPGEIVES(Pi)LS(Pi)S(Pi)S(Pi)EESITR(I)	
1046.58	3	3136.71	3136.72	5	[164-191] (L)SLSQSKVLPVPQKAVPYPQRDMPIQAFL(L)	
1241.01	3	3720.01	3720.03	7	[177-209] (K)AVPYPQRDMPIQAFLLYQEPVLGPVRGPFPIIV(-)	
1494.84	3	4481.48	4481.51	8	[170-209] (K)VLPVPQKAVPYPQRDMPIQAFLLYQEPVLGPVRGPFPIIV(-)	
1121.38	4	4481.48	4481.51	8	[170-209] (K)VLPVPQKAVPYPQRDMPIQAFLLYQEPVLGPVRGPFPIIV(-)	
1278.96	4	5111.79	5111.85	10	[164-209] (L)SLSQSKVLPVPQKAVPYPQRDMPIQAFLLYQEPVLGPVRGPFPIIV(-)	
1415.77	4	5659.05	5659.10	7	[53-105] (F)AQTQSLVYPFPGPIPNSLPQNIPPLTQTPVVVPPFLQPEVLGVSKVKEAMAPK(H)	I
1420.01	4	5676.03	5676.06	6	[53-105] (F)AQTQSLVYPFPGPIPNSLPQNIPPLTQTPVVVPPFLQPEVMGVSKVKEAMAPK(H)	A2
1430.00	4	5715.96	5716.06	6	[53-105] (F)AQTQSLVYPFPGPIHNSLPQNIPPLTQTPVVVPPFLQPEVMGVSKVKEAMAPK(H)	A1

Note：”-” means the sequence terminus.

Table S9 MS/MS fragmentation of the isoform-specific peptide spanning residues 53–105 using higher-energy collisional dissociation (HCD). The observed b- and y-ions confirm amino acid identities at residues 67 and 93 (shown in bold), with m/z values presented as observed (theoretical) for each isoform.

Variant *A2*

1 53 ┌y_{39} ┌y_{13}
RELEELN••• PFAQTQSLVY PFPGPI**P**NSL PQNIPPLTQT PVVVPPFLQP EV**M**GVSKVKE
b_{15}┘ b_{41}┘

105 209
AMAPKHKEMP •••PFPIIV

Variant *A1* (P67→H)

Variant *I* (M93→L)

β-casein Variant	MS1	MS2			
	Residue 53-105 (Da)	Residue 67		Residue 93	
		b_{15}	y_{39}	b_{41}	y_{13}
A1	5715.96 (5716.06)	1635.85 (1635.84)	4217.27 (4217.27)	4472.36 (4472.36)	1374.74 (1374.74)
A2	5676.03 (5676.06)	-	4177.28 (4177.27)	-	1374.74 (1374.74)
I	5659.05 (5659.10)	-	4159.31 (4159.31)	-	1356.78 (1356.78)

Table S10 Peptide fragments from the limited digestion of 2.5 μM GroEL with 1.0 μM trypsin using the nanodroplet platform. Detected sequences are highlighted in red, while undetected regions are shown in black. The identified peptides cover 92% of the GroEL sequence (502 out of 547 amino acids).

1<br>AAKDVKFGND	11<br>ARVKMLRGVN	21<br>VLADAVKVTL	31<br>GPKGRNVVLD	41<br>KSFGAPTITK	51<br>DGVSVAREIE
61<br>LEDKFENMGA	71<br>QMVKEVASKA	81<br>NDAAGDGTTT	91<br>ATVLAQAIIT	101<br>EGLKAVAAGM	111<br>NPMDLKRGID
121<br>KAVTAAVEEL	131<br>KALSVPCSDS	141<br>KAIAQVGTIS	151<br>ANSDETVGKL	161<br>IAEAMDKVGK	171<br>EGVITVEDGT
181<br>GLQDELDVVE	191<br>GMQFDRGYLS	201<br>PYFINKPETG	211<br>AVELESPFIL	221<br>LADKKISNIR	231<br>EMLPVLEAVA
241<br>KAGKPLLIIA	251<br>EDVEGEALAT	261<br>LVVNTMRGIV	271<br>KVAAVKAPGF	281<br>GDRRKAMLQD	291<br>IATLTGGTVI
301<br>SEEIGMELEK	311<br>ATLEDLGQAK	321<br>RVVINKDTTT	331<br>IIDGVGEEAA	341<br>IQGRVAQIRQ	351<br>QIEEATSDYD
361<br>REKLQERVAK	371<br>LAGGVAVIKV	381<br>GAATEVEMKE	391<br>KKARVEDALH	401<br>ATRAAVEEGV	411<br>VAGGGVALIR
421<br>VASKLADLRG	431<br>QNEDQNVGIK	441<br>VALRAMEAPL	451<br>RQIVLNCGEE	461<br>PSVVANTVKG	471<br>GDGNYGYNAA
481<br>TEEYGNMIDM	491<br>GILDPTKVTR	501<br>SALQYAASVA	511<br>GLMITTECMV	521<br>TDLPKNDAAD	531<br>LGAAGGMGGM
541<br>GGMGGMM					

Observed peaks (m/z)	Charge state (z)	Observed mass (Da)	Theoretical mass (Da)	Missed cleavage	Peptide sequence
458.31	1	457.30	457.30	0	[441-444] (K)VALR(A)
533.28	1	532.28	532.29	0	[75-79] (K)EVASK(A)
545.31	1	544.30	544.30	0	[364-367] (K)LQER(V)
586.37	1	585.36	585.36	0	[345-349] (R)VAQIR(Q)
589.34	1	588.34	587.34	1	[117-121] (K)RGIDK(A)
614.39	1	613.38	613.38	0	[28-33] (K)VTLGPK(G)
631.37	1	630.36	630.37	1	[1-6] (-)AAKDVK(F)
679.32	1	678.31	678.31	0	[7-12] (K)FGNDAR(V)
703.38	1	702.37	702.37	0	[51-57] (K)DGVSVAR(E)
787.42	1	786.41	786.41	0	[445-451] (R)AMEAPLR(Q)
827.54	1	826.54	826.53	0	[371-379] (K)LAGGVAVIK(V)
985.55	1	984.54	984.56	0	[18-27] (R)GVNVLADAVK(V)
1011.53	1	1010.53	1010.51	0	[395-403] (R)VEDALHATR(A)
506.26	2	1010.51	1010.51	0	[395-403] (R)VEDALHATR(A)
563.38	2	1124.74	1124.73	1	[368-379] (R)VAKLAGGVAVIK(V)
594.34	2	1186.65	1186.65	1	[272-283] (K)VAAVKAPGFGDR(R)
601.33	2	1200.65	1200.65	1	[311-321] (K)ATLEDLGQAKR(V)
619.84	2	1237.66	1237.65	1	[393-403] (K)ARVEDALHATR(A)
1291.63	1	1290.62	1290.65	1	[380-391] (K)VGAATEVEMKEK(K)
646.33	2	1290.65	1290.67	2	[1-12] (-)AAKDVKFGNDAR(V)
672.39	2	1342.76	1342.75	2	[272-284] (K)VAAVKAPGFGDRR(K)
683.88	2	1365.75	1365.75	2	[392-403] (K)KARVEDALHATR(A)
687.35	2	1372.69	1372.69	1	[105-117] (K)AVAAGMNPMDLKR(G)
693.41	2	1384.80	1384.79	1	[15-27] (K)MLRGVNVLADAVK(V)
714.75	2	1427.48	1418.74	2	[380-392] (K)VGAATEVEMKEKK(A)
722.40	2	1442.77	1442.80	1	[118-131] (R)GIDKAVTAAVEELK(A)
727.82	2	1453.63	1453.63	0	[350-361] (R)QQIEEATSDYDR(E)
784.45	2	1566.89	1566.87	0	[404-420] (R)AAVEEGVVAGGGVALIR(V)
790.98	2	1579.94	1579.93	1	[18-33] (R)GVNVLADAVKVTLGPK(G)
795.45	2	1588.88	1588.88	1	[36-50] (R)NVVLDKSFGAPTITK(D)
803.43	2	1604.84	1604.85	1	[42-57] (K)SFGAPTITKDGVSVAR(E)
820.93	2	1639.84	1639.86	1	[430-444] (R)GQNEDQNVGIKVALR(A)
856.40	2	1710.78	1710.77	1	[350-363] (R)QQIEEATSDYDREK(L)

(Table continues on the following page)

880.96	2	1759.91	1759.89	0	[142-159] (K)AIAQVGTISANSDETVGK(L)
885.47	2	1768.92	1768.91	1	[425-440] (K)LADLRGQNEDQNVGIK(V)
922.03	2	1842.04	1842.03	1	[371-389] (K)LAGGVAVIKVGAATEVEMK(E)
623.70	3	1868.08	1868.11	4	[268-285] (R)GIVKVAAVKAPGFGDRRK(A)
950.51	2	1899.00	1898.98	0	[452-469] (R)QIVLNCGEEPSVVANTVK(G)
964.88	2	1927.74	1927.73	0	[526-547] (K)NDAADLGAAGGMGGMGGMGGMM(-)
991.07	2	1980.12	1980.15	2	[15-33] (K)MLRGVNVLADAVKVTLGPK(G)
1005.99	2	2009.96	2009.94	1	[58-74] (R)EIELEDKFENMGAQMVK(E)
1009.54	2	2017.07	2017.04	1	[122-141] (K)AVTAAVEELKALSVPCSDSK(A)
1050.60	2	2099.19	2099.17	2	[371-391] (K)LAGGVAVIKVGAATEVEMKEK(K)
714.42	3	2140.22	2140.23	2	[368-389] (R)VAKLAGGVAVIKVGAATEVEMK(E)
1078.08	2	2154.15	2154.14	2	[421-440] (R)VASKLADLRGQNEDQNVGIK(V)
719.06	3	2154.15	2154.14	2	[421-440] (R)VASKLADLRGQNEDQNVGIK(V)
1105.12	2	2208.22	2208.20	2	[425-444] (K)LADLRGQNEDQNVGIKVALR(A)
737.08	3	2208.21	2208.20	2	[425-444] (K)LADLRGQNEDQNVGIKVALR(A)
743.44	3	2227.28	2227.26	3	[371-392] (K)LAGGVAVIKVGAATEVEMKEKK(A)
1119.55	2	2237.08	2237.06	2	[350-367] (R)QQIEEATSDYDREKLQER(V)
746.70	3	2237.07	2237.06	2	[350-367] (R)QQIEEATSDYDREKLQER(V)
1137.64	2	2273.25	2273.24	2	[36-57] (R)NVVLDKSFGAPTITKDGVSVAR(E)
1200.16	2	2398.30	2398.27	1	[322-344] (R)VVINKDTTTIIDGVGEEAAIQGR(V)
800.45	3	2398.31	2398.27	1	[322-344] (R)VVINKDTTTIIDGVGEEAAIQGR(V)
1216.16	2	2430.30	2430.27	2	[118-141] (R)GIDKAVTAAVEELKALSVPCSDSK(A)
811.10	3	2430.28	2430.27	2	[118-141] (R)GIDKAVTAAVEELKALSVPCSDSK(A)
829.80	3	2486.37	2486.36	3	[34-57] (K)GRNVVLDKSFGAPTITKDGVSVAR(E)
1263.14	2	2524.26	2524.22	2	[58-79] (R)EIELEDKFENMGAQMVKEVASK(A)
842.41	3	2524.21	2524.22	2	[58-79] (R)EIELEDKFENMGAQMVKEVASK(A)
846.09	3	2535.24	2535.26	3	[350-370] (R)QQIEEATSDYDREKLQERVAK(L)
852.47	3	2554.40	2554.37	2	[321-344] (K)RVVINKDTTTIIDGVGEEAAIQGR(V)
863.13	3	2586.38	2586.37	3	[117-141] (K)RGIDKAVTAAVEELKALSVPCSDSK(A)
865.49	3	2593.43	2593.43	3	[421-444] (R)VASKLADLRGQNEDQNVGIKVALR(A)
878.13	3	2631.36	2631.34	1	[142-167] (K)AIAQVGTISANSDETVGKLIAEAMDK(V)
1325.19	2	2648.36	2648.33	0	[286-310] (K)AMLQDIATLTGGTVISEEIGMELEK(A)
883.79	3	2648.35	2648.33	0	[286-310] (K)AMLQDIATLTGGTVISEEIGMELEK(A)
1334.70	2	2667.39	2667.37	1	[445-469] (R)AMEAPLRQIVLNCGEEPSVVANTVK(G)
890.12	3	2667.34	2667.37	1	[445-469] (R)AMEAPLRQIVLNCGEEPSVVANTVK(G)
1362.27	2	2722.52	2722.49	0	[242-267] (K)AGKPLLIIAEDVEGEALATLVVNTMR(G)
908.51	3	2722.52	2722.49	0	[242-267] (K)AGKPLLIIAEDVEGEALATLVVNTMR(G)
926.49	3	2776.46	2776.42	1	[285-310] (R)KAMLQDIATLTGGTVISEEIGMELEK(A)
935.82	3	2804.44	2804.40	3	[345-367] (R)VAQIRQQIEEATSDYDREKLQER(V)
1458.73	2	2915.45	2915.51	1	[75-104] (K)EVASKANDAAGDGTTTATVLAQAIITEGLK(A)
972.86	3	2915.56	2915.53	2	[142-170] (K)AIAQVGTISANSDETVGKLIAEAMDKVGK(E)
978.51	3	2932.50	2932.52	2	[284-310] (R)RKAMLQDIATLTGGTVISEEIGMELEK(A)
989.44	3	2965.30	2965.27	0	[470-497] (K)GGDGNYGYNAATEEYGNMIDMGILDPTK(V)
1037.89	3	3110.66	3110.62	0	[197-224] (R)GYLSPYFINKPETGAVELESPFILLADK(K)
1661.75	2	3321.49	3321.49	1	[470-500] (K)GGDGNYGYNAATEEYGNMIDMGILDPTKVTR(S)
1108.16	3	3321.46	3321.49	1	[470-500] (K)GGDGNYGYNAATEEYGNMIDMGILDPTKVTR(S)
1225.96	3	3674.84	3674.86	1	[286-320] (K)AMLQDIATLTGGTVISEEIGMELEKATLEDLGQAK(R)
1252.97	3	3755.90	3755.92	2	[80-117] (K)ANDAAGDGTTTATVLAQAIITEGLKAVAAGMNPMDLKR(G)
939.99	4	3755.92	3755.92	2	[80-117] (K)ANDAAGDGTTTATVLAQAIITEGLKAVAAGMNPMDLKR(G)
1268.64	3	3802.90	3802.96	2	[285-320] (R)KAMLQDIATLTGGTVISEEIGMELEKATLEDLGQAK(R)
1277.99	3	3830.94	3830.96	2	[286-321] (K)AMLQDIATLTGGTVISEEIGMELEKATLEDLGQAKR(V)
1320.68	3	3959.01	3959.06	3	[284-320] (R)RKAMLQDIATLTGGTVISEEIGMELEKATLEDLGQAK(R)
1320.68	3	3959.01	3959.06	3	[285-321] (R)KAMLQDIATLTGGTVISEEIGMELEKATLEDLGQAKR(V)
1372.70	3	4115.08	4115.16	4	[284-321] (R)RKAMLQDIATLTGGTVISEEIGMELEKATLEDLGQAKR(V)
1424.40	3	4270.17	4270.19	3	[75-117] (K)EVASKANDAAGDGTTTATVLAQAIITEGLKAVAAGMNPMDLKR(G)
1508.35	3	4522.02	4522.01	1	[501-547] (R)SALQYAASVAGLMITTECMVTDLPKNDAADLGAAGGMGGMGGMGGMM(-)
1301.61	4	5202.42	5202.46	2	[452-500] (R)QIVLNCGEEPSVVANTVKGGDGNYGYNAATEEYGNMIDMGILDPTKVTR(S)

Note：”-” means the sequence terminus.

Table S11 Twelve proteolytic peptides (probability score > 0.99) were identified for the GroEL protein complex (accession number: A1AJ51) by peptide-level DDA-MS analysis.

Peptide	Charges
[19-28] GVNVLADAVK	1
[59-75] EIELEDKFENMGAQMVK	2
[81-105] ANDAAGDGTTTATVLAQAIITEGLK	2, 3
[161-168] LIAEAMDK	1
[172-197] EGVITVEDGTGLQDELDVVEGMQFDR	2, 3
[243-268] AGKPLLIIAEDVEGEALATLVVNTMR	2, 3
[286-311] KAMLQDIATLTGGTVISEEIGMELEK	2
[287-311] AMLQDIATLTGGTVISEEIGMELEK	2
[351-362] QQIEEATSDYDR	2
[372-380] LAGGVAVIK	1
[446-452] AMEAPLR	1
[471-498] GGDGNYGYNAATEEYGNMIDMGILDPTK	2
[527-548] NDAADLGAAGGMGGMGGMGGMM	2

Table S12 Thirteen proteolytic peptides (probability score > 0.99) were identified for ArnA (accession number: B1IXT2) by peptide-level DDA-MS analysis.

Peptide	Charges
[86-110] HLIYDEILQLAPAGAFNLHGSLLPK	3
[113-134] GRAPLNWVLVNGETETGVTLHR	2, 3
[115-134] APLNWVLVNGETETGVTLHR	3
[138-149] RADAGAIVAQLR	2
[171-181] QLLEQTLPAIK	2
[171-191] QLLEQTLPAIKHGNILEIAQR	3
[182-191] HGNILEIAQR	2
[201-222] RTPDDSFLEWHKPASVLHNMVR	3
[202-222] TPDDSFLEWHKPASVLHNMVR	3
[223-239] AVADPWPGAFSYVGNQK	2
[318-334] VLILGVNGFIGNHLTER	2
[405-415] VFELDFEENLR	2
[636-651] IDMQETIDETLDFFLR	2

Table S13 Reproducibility of nanodroplet digestion under limited-digestion conditions was evaluated across replicate experiments. Consistency was assessed based on peptide identifications, sequence coverage, and coexistence of intact protein and digestion-derived peptide signals.

Replicate	Intact protein observed	Identified peptides	Sequence coverage (%)
1	Yes	36	84
2	Yes	36	84
3	Yes	33	84

Table S14 Effect of acquisition time on signal-to-noise ratio (S/N), peptide identification, and sequence coverage for nanodroplet digestion of myoglobin under native-compatible conditions.

Acquisition time	Low-intensity peptide S/N†	High-intensity peptide S/N‡	Number of identified peptides	Sequence coverage (%)
10 s	65	1645	35	84
60 s	200	3265	35	84
180 s	450	3600	36	84

†Estimated from a representative low-intensity peptide signal (~1% relative intensity) at m/z 831.45.

‡Estimated from a representative high-intensity peptide signal at m/z 1135.29.
